# Probing ligand recognition of the opioid pan antagonist AT-076 at nociceptin, kappa, mu, and delta opioid receptors through structure-activity relationships

**DOI:** 10.1038/s41598-017-13129-1

**Published:** 2017-10-16

**Authors:** V. Blair Journigan, Willma E. Polgar, Edward W. Tuan, James Lu, Pankaj R. Daga, Nurulain T. Zaveri

**Affiliations:** 1 Astraea Therapeutics, 320 Logue Avenue, Suite 142, Mountain View, CA 94043 USA; 2 Present Address: Marshall University School of Pharmacy, Department of Pharmaceutical Sciences, One John Marshall Drive, Huntington, WV 25755 USA

## Abstract

Few opioid ligands binding to the three classic opioid receptor subtypes, mu, kappa and delta, have high affinity at the fourth opioid receptor, the nociceptin/orphanin FQ receptor (NOP). We recently reported the discovery of **AT-076** (**1**), (R)-7-hydroxy-N-((S)-1-(4-(3-hydroxyphenyl)piperidin-1-yl)-3-methylbutan-2-yl)-1,2,3,4-tetrahydroisoquinoline-3-carboxamide, a pan antagonist with nanomolar affinity for all four subtypes. Since AT-076 binds with high affinity at all four subtypes, we conducted a structure-activity relationship (SAR) study to probe ligand recognition features important for pan opioid receptor activity, using chemical modifications of key pharmacophoric groups. SAR analysis of the resulting analogs suggests that for the NOP receptor, the entire AT-076 scaffold is crucial for high binding affinity, but the binding mode is likely different from that of NOP antagonists C-24 and SB-612111 bound in the NOP crystal structure. On the other hand, modifications of the 3-hydroxyphenyl pharmacophore, but not the 7-hydroxy Tic pharmacophore, are better tolerated at kappa and mu receptors and yield very high affinity multifunctional (e.g. **12**) or highly selective (e.g. **16**) kappa ligands. With the availability of the opioid receptor crystal structures, our SAR analysis of the common chemotype of AT-076 suggests rational approaches to modulate binding selectivity, enabling the design of multifunctional or selective opioid ligands from such scaffolds.

## Introduction

**Figure 1:**
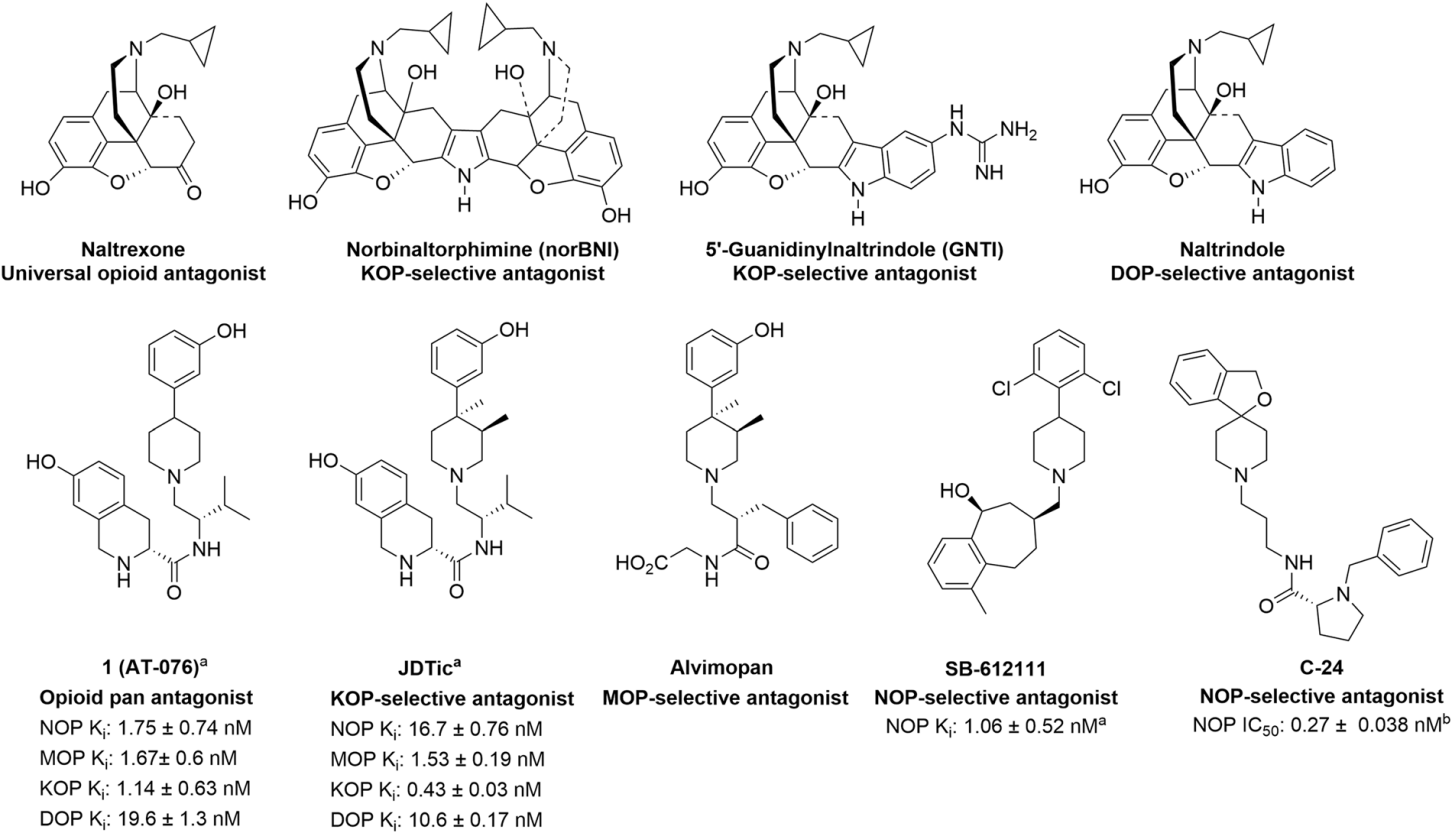
Morphinan-type (upper row) and nonmorphinan-type (lower row) phenylpiperidine-containing opioid antagonists. ^a^From ref.^15^. ^b^From ref.^16^.

Very few opioid ligands show promiscuous high-affinity binding to all four opioid receptor subtypes, mu, kappa, delta and the nociceptin opioid receptors (MOP, KOP, DOP, NOP respectively). In fact, it is well documented in the literature that the most opioid ligands which have high affinity for the three classic opioid receptors, MOP, KOP and DOP, have little to no affinity for the NOP receptor^1–3^. Prior to the recent determination of the X-ray crystal structures of the four opioid receptors bound to their selective antagonist ligands, elegant structure-activity relationship (SAR) studies of opioid ligands, in conjunction with site-directed mutagenesis, provided seminal information on the similarities and differences in opioid receptor binding pockets and selectivity-enhancing pharmacophoric features of opioid ligands. Using these approaches receptor-selective opioid ligands were designed from universal opioid scaffolds; for example, kappa-selective antagonist norbinaltorphimine (norBNI)^4,5^ and delta-selective antagonist naltrindole (NTI)^6^ were designed from the non-selective opioid antagonist naltrexone (Fig. 1), and the kappa-selective antagonist, 5′-guanidinylnaltrindole (GNTI) was designed from the delta-selective antagonist NTI^7,8^. Binding modes of these antagonists in the opioid receptor homology-based models were derived by docking a universal opioid antagonist such as naltrexone as the ‘common pharmacophore’ or ‘message’ into the opioid binding pocket and refined based on the observed SAR of these ligands and the message-address concept^9^. The selectivity of the various naltrexone-derived antagonists was explained by the orientation and interaction of the ‘address’ elements of these ligands with different amino acid residues in the ligand-binding pocket, viz. the address domains of the opioid receptors^10^. These binding models were further confirmed by site-directed mutagenesis studies^11,12^, and, together with the SAR and docking studies, provided a sound understanding of the structural and molecular basis of ligand recognition at the opioid receptors, even before the ligand-bound opioid receptor crystal structures were elucidated. Notably, the DOP crystal structure bound to antagonist naltrindole^13^ and the MOP crystal structure bound to antagonist β-FNA^14^, show that the binding orientation of these antagonists are consistent with binding models previously proposed based on the opioid homology models^10,12^. The discoveries of highly selective opioid tool ligands from common opioid chemotypes like the morphinans underscore the importance of SAR and receptor structure-based rational chemical modifications to the field of opioid ligand drug design.

We recently reported an opioid antagonist AT-076 (**1**), which has nanomolar affinity for all four opioid receptor subtypes^15^. This opioid pan-antagonist is a non-morphinan opioid ligand, containing a phenylpiperidine scaffold and is a close analog of the kappa-selective antagonist JDTic (Fig. 1). The phenylpiperidine moiety in **1** and the (3 R,4 R)-dimethyl-4-(3-hydroxyphenyl)piperidine scaffold in JDTic are common nonmorphinan opioid antagonist pharmacophores, present in other opioid antagonists such as the mu opioid-selective antagonist alvimopan, (Fig. 1) and the NOP antagonists C-24 and SB-612111 (Fig. 1).

The nanomolar binding affinity of AT-076 to all four opioid receptors suggests that AT-076 possesses a chemotype that can bind with high affinity at all four opioid receptors and can function as a universal opioid scaffold. We therefore conducted a SAR study to probe the chemical features of AT-076 that play a role in ligand recognition at the four opioid receptors.

**Figure 2:**
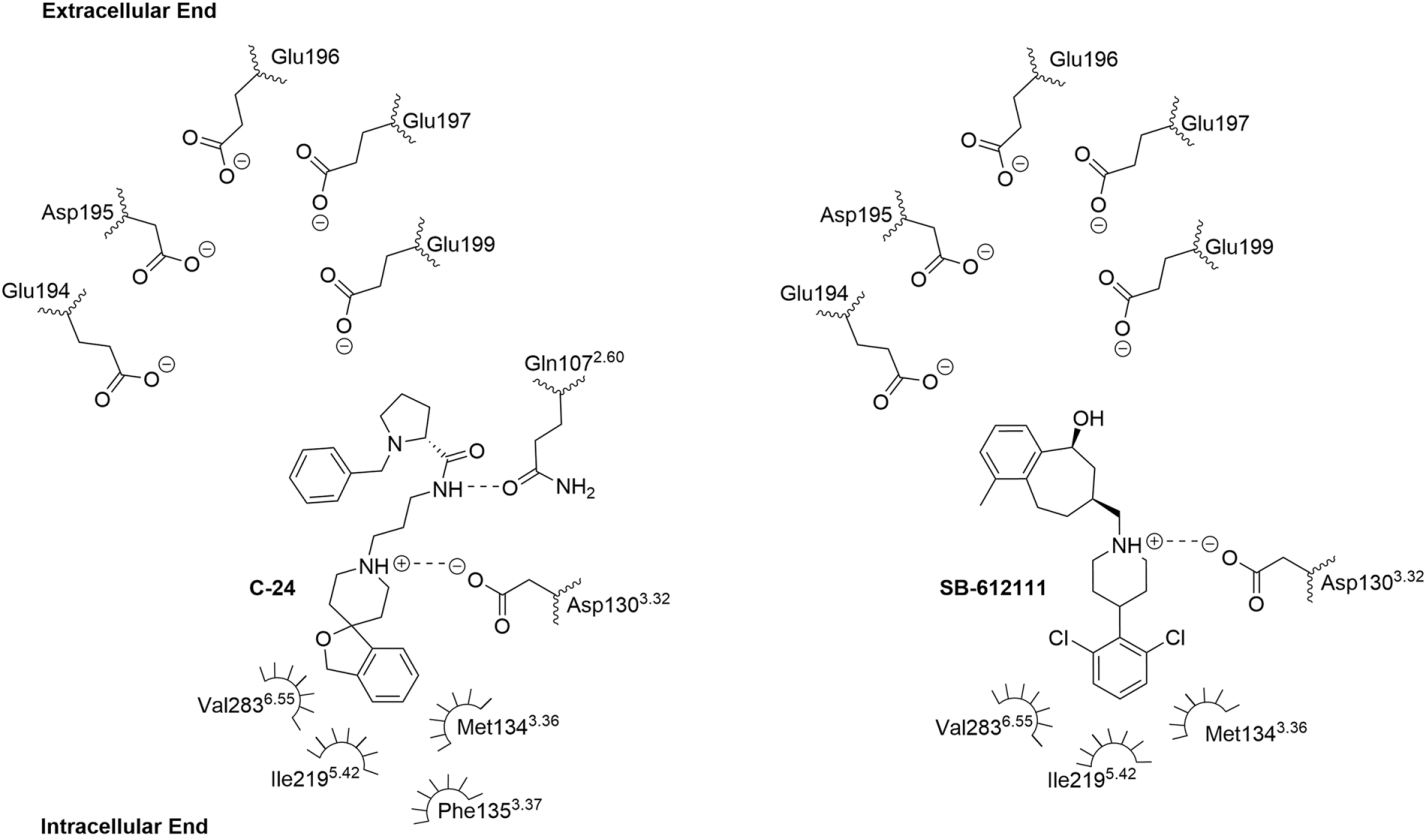
Bound orientation of C-24 and SB-612111 in the NOP receptor x-ray crystal structure.

AT-076, being a phenylpiperidine-based non-morphinan opioid antagonist, is a close structural analog of the nonmorphinan kappa antagonist JDTic and similar to the phenylpiperidine-based NOP antagonists C-24 and SB-612111 (Fig. 1). Previously, we reported docking models of AT-076 in the KOP and NOP crystal structures (PDB No: 4DJH^17^ and PDB No: 4EA3^18^ respectively), which provided putative binding orientations of AT-076 in the NOP and KOP receptors^15^. The highest-scoring docked orientation of AT-076 in the NOP binding pocket was similar to the binding orientations of crystallized NOP antagonists C-24 and SB-612111 in the NOP receptor (shown in Fig. 2), such that the aromatic moiety at the 4-position of the piperidine ring (benzofuran ring in C-24, 2,6-dichlorophenyl in SB-612111, and 3-hydroxyphenyl in AT-076) was oriented towards the intracellular end of the binding pocket, consisting of hydrophobic residues Met134^3.36^, Phe135^3.37^, Ile219^5.42^ and Val283^6.55^.

**Figure 3:**
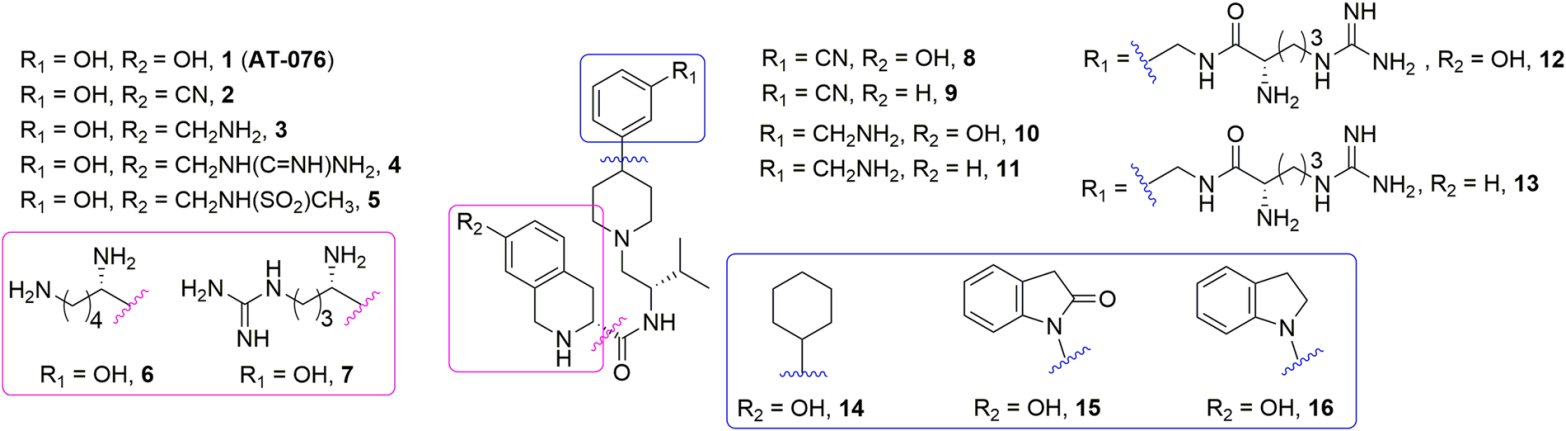
Modifications of **AT-076** to explore the SAR at the four opioid receptors. Target compound numbers are indicated in **bold**.

A related aim of our study was to confirm these putative docking orientations through an SAR analysis of AT-076 by introducing rational chemical modifications based on the putative docking poses of AT-076 at the NOP and KOP receptors. Figure 3 shows the structures of analogs **2**–**16** designed to provide information on the ligand recognition features of AT-076 important for providing high affinity at the four opioid receptors.

We previously reported that the 7-hydroxy group on the tetrahydroisoquinolinyl (Tic) moiety of AT-076 was important for maintaining high binding affinity at all four opioid receptors, because removal of this hydroxy group significantly decreased affinity at all four opioid receptors^15^. The 3-hydroxy group on the 4-phenylpiperidine moiety however, could be removed with a small loss in binding affinity at the NOP, MOP and KOP, but caused a loss in affinity at DOP receptors. We focused our SAR modifications on the 7-hydroxy and 3-hydroxy groups of AT-076 to obtain more information on the binding orientations of AT-076 in the opioid receptors, given the known differences in amino acid residues in the transmembrane domains and binding pockets of the four opioid receptors^19^.

Both the NOP and KOP receptors (but not DOP and MOP) contain anionic amino acid residues in their extracellular loop (EL) 2 (between transmembrane helices (TM) 4 and 5), which have been shown to function as the address domains for these receptors and interact with the cationic core residues of their respective endogenous peptide ligands nociceptin and dynorphin^20–23^. For kappa antagonists such as norBNI and 5′-GNTI, the selectivity-enhancing ‘address’ interaction occurs with the nonconserved residue Glu297^6.58^ at the extracellular end of TM6^8,10–12^. To explore and confirm binding orientations of AT-076, we introduced various positively charged groups in the 7-hydroxy-Tic moiety by replacing the 7-hydroxy group with amine (**3**), guanidine (**4**) and N-methyl sulfonamide (**5**) groups; as well as substitution of the Tic-OH heterocycle with lysine (**6**) and arginine (**7**). For comparison with these charged analogs, the 7-OH was also replaced with a cyano group (**2**). Similarly, the 3-hydroxy group of the phenylpiperidine moiety was also replaced with a cyano (**8**), amine (**10**) and arginine groups (**12**). Additionally, for these compounds, the effect of removal of the 7-OH of the Tic moiety was also explored (**9**, **11** and **13**, respectively).

As shown in the 2D diagram in Fig. 2, derived from the C-24 and SB-612111-bound NOP receptor crystal structures^18,24^, the benzofuran and 2,6-dichlorophenyl moieties at the 4-position of the piperidine are oriented towards the intracellular end of the TM binding pocket, surrounded by hydrophobic residues Met134^3.36^, Phe135^3.37^, Ile219^5.42^ and Val283^6.55^. Docking of AT-076 in the NOP crystal structure also resulted in a similar orientation of the piperidine ring, where the 3-hydroxyphenyl ring at the 4-position of the piperidine ring was oriented towards this hydrophobic domain towards the intracellular end of the TM binding pocket^15^. In order to confirm such an orientation of AT-076, we replaced the 3-hydroxyphenyl group with other hydrophobic moieties such as the cyclohexyl (**14**), indolinone (**15**) as well as an indoline ring (**16**). The synthesis of the resulting analogs, their detailed *in vitro* pharmacological characterization and SAR analysis is discussed below.

## Chemistry

**Figure 4:**
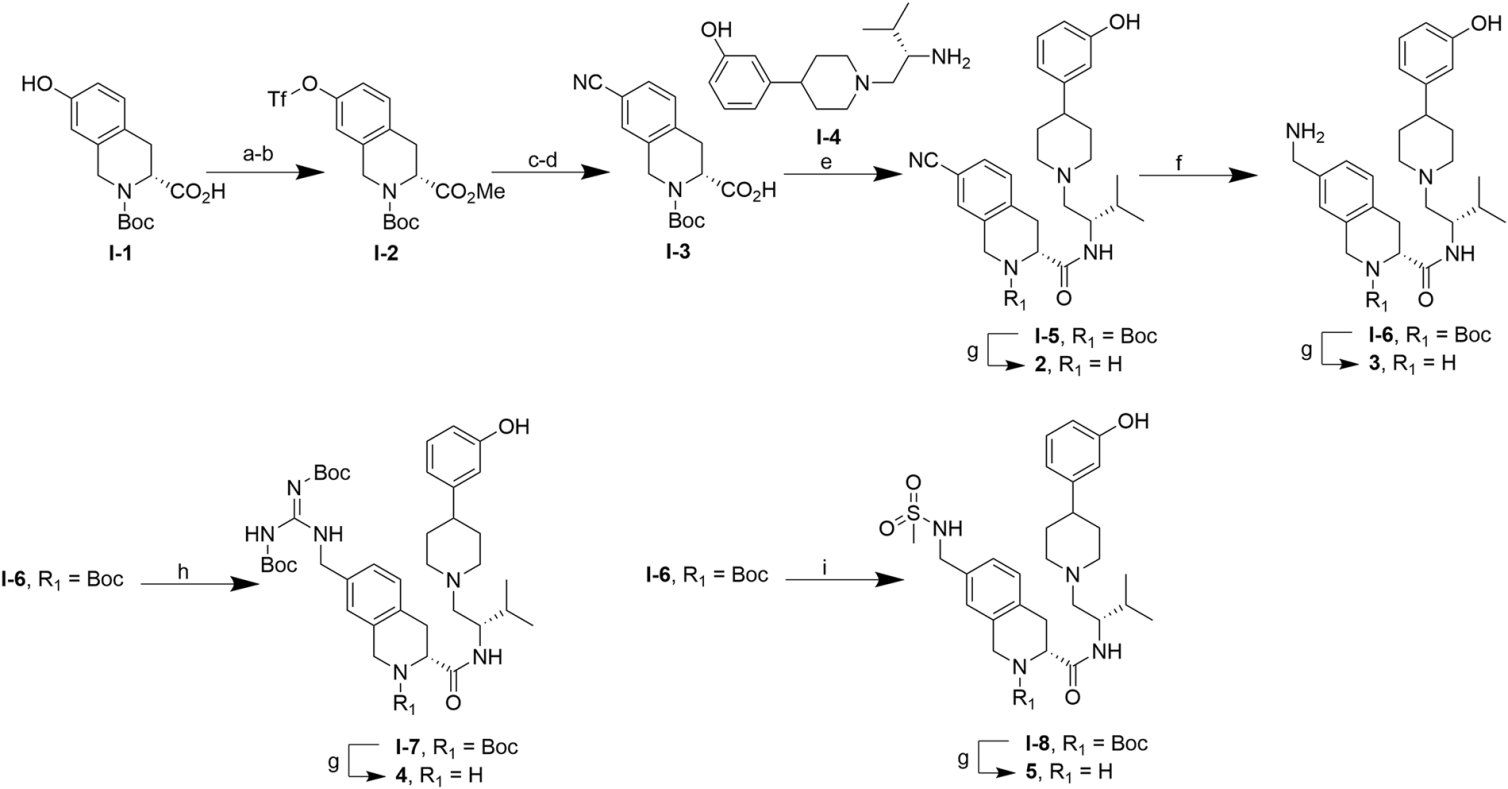
Synthesis of **2–5**. ***Reagents and conditions:*** (**a**) LiOH, Me_2_SO_4_, acetone, reflux, quantitative; (**b**) N-phenyl-bis(trifluoromethanesulfonimide), K_2_CO_3_, THF, 22 h, rt, 86%; (**c**) ZnCN_2_,Pd(PPh_3_)_4_, DMF, 16 h, 100 °C, 86%; (**d**) LiOH, THF, 1 h, rt, quantitative; (**e**) BOP, Et_3_N, THF, 19 h, rt, 74%; (**f**) Raney Ni, 4.8 atm H_2_(g), NH_3_/MeOH, 21 h, rt, 99%; (**g**) HCl/dioxane, MeOH, 2–6 h, rt, 40%-quantitative; (**h**) 1,3-bis (*tert*-butoxycarbonyl)-2-methyl-2-thiopseudourea, HgCl_2_, Et_3_N, THF, 2.5 h, rt, 36%, (**i**) Cl-SO_2_CH_3_, pyridine, CH_2_Cl_2_, 16 h, rt, 50%.

Cyano (**2**), amine (**3**), guanidine (**4**) and N-methyl sulfonamide (**5**) substitutions of the 7-hydroxy Tic-OH were prepared as shown in Fig. 4. Selective methylation of (3 R)-2-(tert-butoxycarbonyl)-7-hydroxy-1,2,3,4-tetrahydroisoquinoline-3-carboxylic acid **I-1** was achieved in quantitative yield using LiOH and dimethyl sulfate^25^, followed by treatment of the free phenol with N-phenyl-bis(trifluoromethanesulfonimide) (PhNTf_2_)^26^ to triflate **I-2**. Subsequent Pd-catalyzed cyanation^27^ and LiOH deprotection afforded **I-3**. BOP-mediated amidation of carboxylic acid **I-3** with amine (S)-3-(1-(2-amino-3-methylbutyl)piperidin-4-yl)phenol **I**-**4** (prepared according to literature methods)^15^ gave **I-5**. HCl deprotection then afforded **2**. Hydrogenation of the nitrile of **I-5** with Raney Ni yielded the amine **I-6**, which upon removal of the Boc group with HCl afforded **3**. Amine **I-6** was subjected to HgCl_2_-mediated guanidation using 1,3-bis (*tert*-butoxycarbonyl)-2-methyl-2-thiopseudourea to give intermediate **I-7** ^28^, and mono-sulfonated with methylsulfonyl chloride/pyridine^29,30^ to give intermediate **I-8**. HCl deprotection of intermediates **I-7** and **I-8** afforded **4** and **5**, respectively.

**Figure 5:**
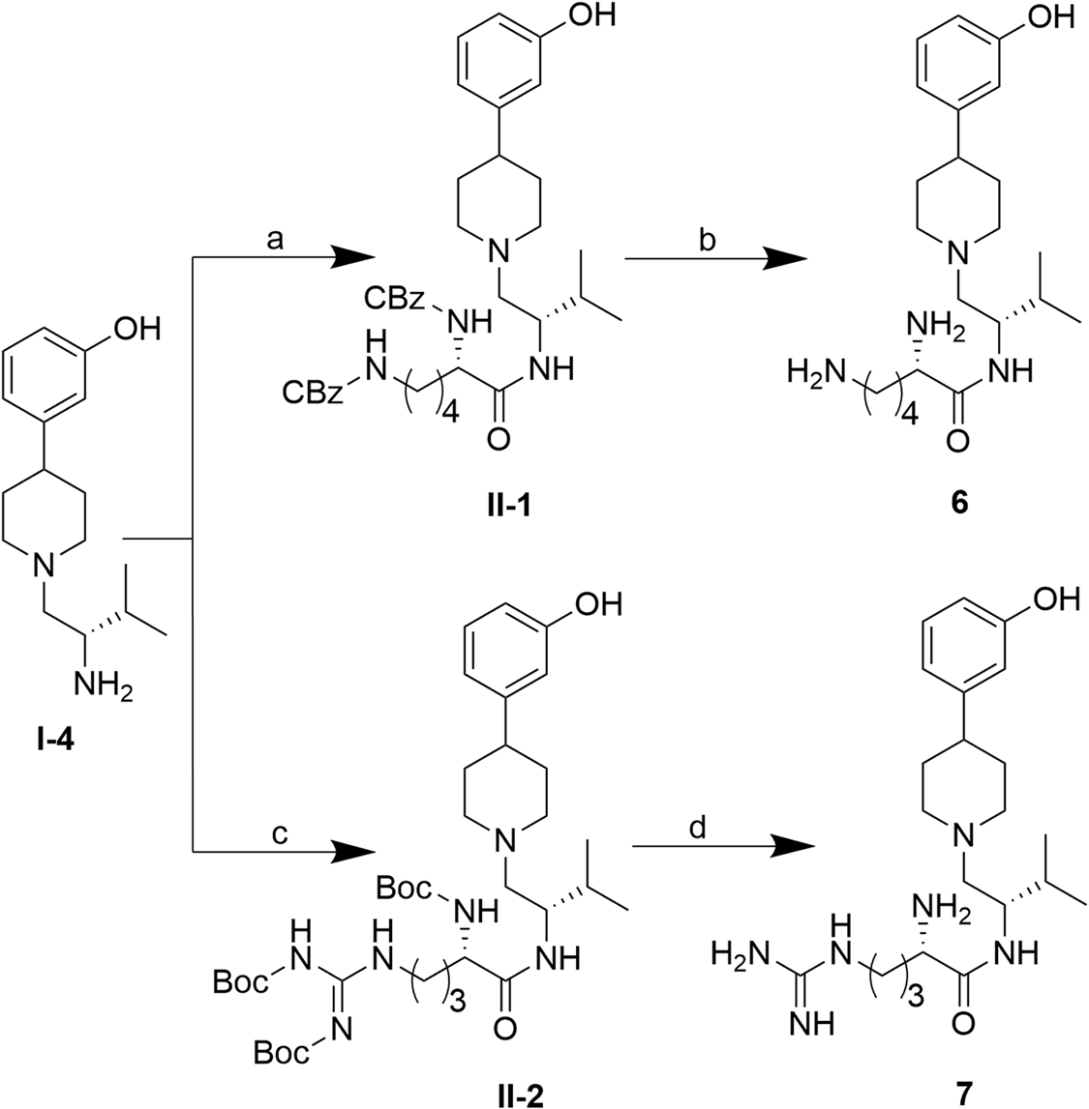
Synthesis of **6**–**7**. ***Reagents and conditions***: (**a**) N2,N6-Bis(benzyloxycarbonyl)-L-lysine, BOP, Et_3_N, THF, 3.5 h, rt, 64%; (**b**) 1 atm H_2_(g), Pd/C 10%, THF, 6 h, rt, quantitative; (**c**) Boc-Arg(Boc)_2_-OH, BOP, Et_3_N, THF, 2 h, rt, 95%; (**d**) TFA, CH_2_Cl_2_, 2 h, rt, 79%.

Analogs bearing positively charged replacements of the Tic-OH, **6** and **7**, were synthesized as shown in Fig. 5. BOP-mediated coupling of amine **I**-**4** ^15^ and N2,N6-bis(benzyloxycarbonyl)-L-lysine or Boc-Arg(Boc)_2_-OH afforded amides **II**-**1** and **II**-**2**, respectively. Hydrogenolysis of CBz-protected **II**-**1** and trifluoroacetic acid cleavage of Boc-protected **II**-**2**, afforded final products **6** and **7**, respectively.

**Figure 6:**
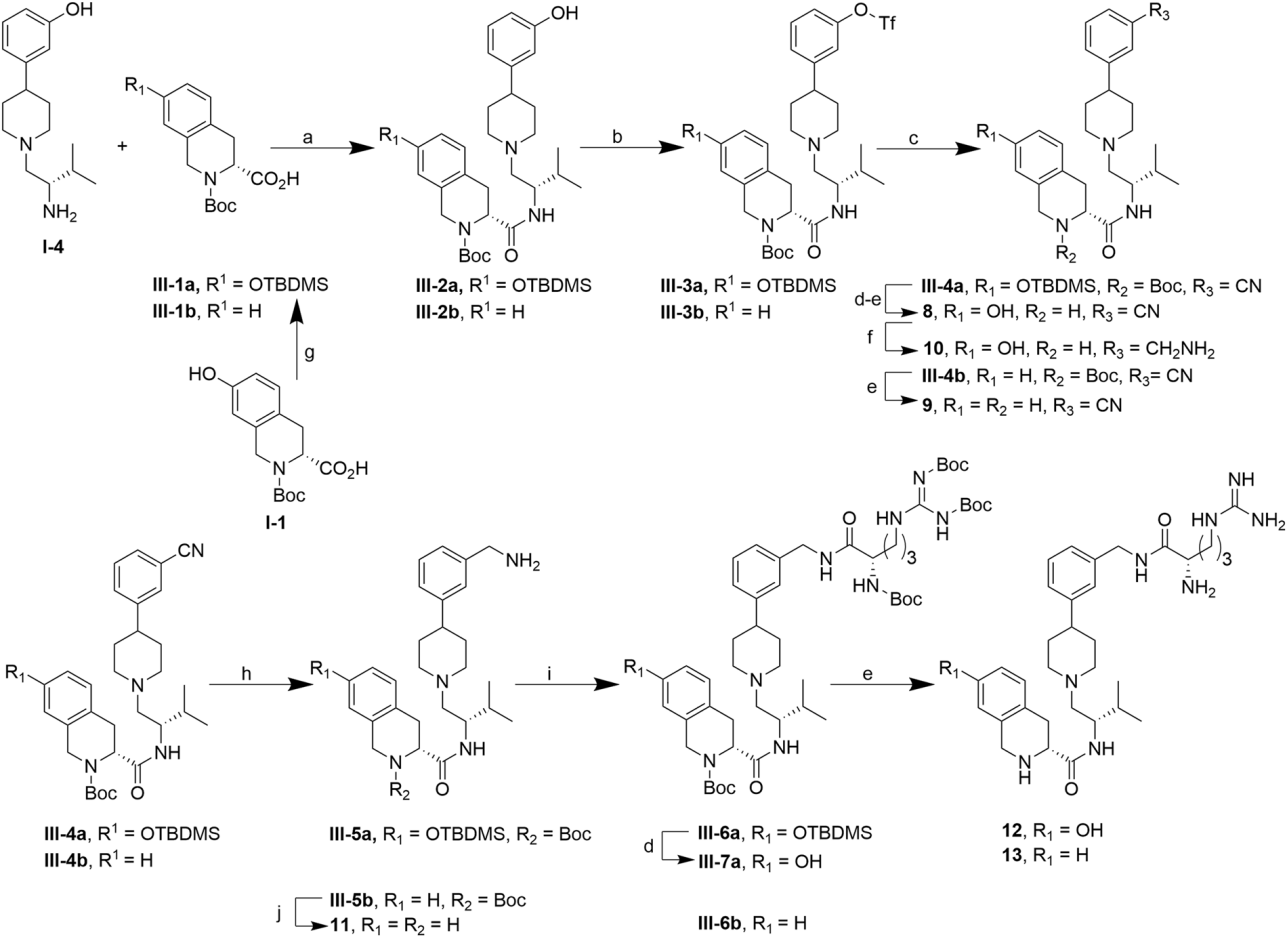
Synthesis of **8**–**13**. ***Reagents and conditions***: (**a**) BOP, Et_3_N, THF, 5 h, rt, 87%; (**b**) N-phenyl-bis(trifluoromethanesulfonimide), K_2_CO_3_, THF, 18–26 h, rt, 50–63%; (**c**) ZnCN_2_, Pd(PPh_3_)_4_, DMF, 1 h, 80 °C, 67–91%; (**d**) TBAF/THF, THF, 1–2 h, rt, 53%-quantitative; (**e**) TFA, CH_2_Cl_2_, 1–5.5 h, rt, 58–93%; (**f**) Raney Ni, 4.8 atm H_2_(g), NH_3_/MeOH, 19 h, rt, 88%; (**g**) TBDMS-Cl, imidazole, DMF, 3 h, rt, 82%; (**h**) Raney Ni, 4–4.8 atm H_2_(g), NH_3_/MeOH, 5.5 h, rt, 64–70%; (**i**) Boc-Arg(Boc)_2_-OH, EDCI, HOBt, Et_3_N, CH_2_Cl_2_ or DMF, 24 h, rt, 46–58%; (**j**) HCl/ether, MeOH, 19 h, rt, 89%.

Cyano, amine and arginine substitutions (**8**, **10** and **12**, respectively) of the 3-hydroxy of the phenylpiperidine, as well as corresponding analogs lacking the 7-hydroxyl substitution at the Tic moiety (**9**, **11**, **13**, respectively), were synthesized as shown in Fig. 6. For the cyano analog **8**, amine **I**-**4** ^15^ was first coupled with TBDMS-protected carboxylic acid **III**-**1a** using standard BOP-mediated coupling to amide **III**-**2a**. Corresponding analog **III**-**2b**, lacking the 7-hydroxyl at the Tic moiety, was prepared as previously reported^15^. Conversion of the 3-hydroxyl on the phenylpiperidine of **III-2a** and **III-2b** to triflates **III**-**3a** and **III-3b** using N-phenyl-bis(trifluoromethanesulfonimide) (PhNTf_2_)^26^, followed by Pd-catalyzed cyanation furnished **III**-**4a** and **III-4b** in good yields^27^. TBDMS- and Boc-deprotection of **III**-**4a** afforded cyano analog **8** in reasonable overall yield. Reduction of **8** with Raney Ni/H_2_(g) then afforded **10**. Boc deprotection of **III**-**4b** with trifluoroacetic acid yielded cyano analog **9**. To obtain analogs **11–13**, protected cyano intermediates **III-4a** and **III-4b** were reduced to their corresponding amines **III-5a** and **III-5b** using Raney Ni/H_2_(g). Boc deprotection of **III-5b** with HCl furnished **11**. EDCI/HOBt-mediated coupling of amines **III-5a** and **III-5b** with Boc-Arg(Boc)_2_-OH, followed by TBDMS- and Boc-deprotection afforded arginine analogs **12** and **13**.

**Figure 7:**
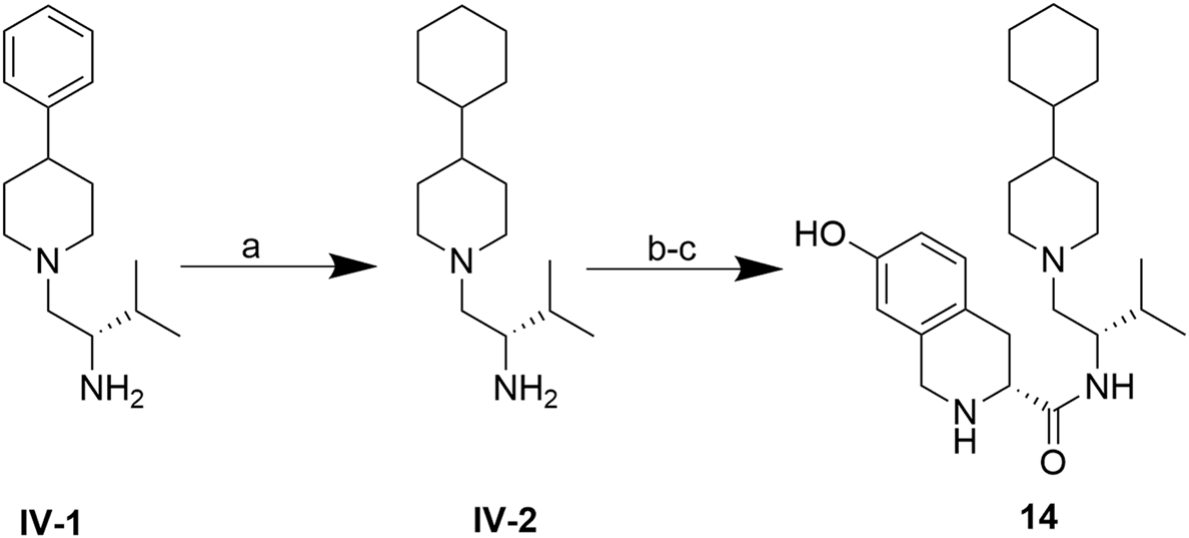
Synthesis of **14**. ***Reagents and conditions***: (**a**) PtO_2_, 4 atm H_2_(g), concd. HCl (aq), MeOH, 2.5 h, rt, quantitative; (**b**) **I-1**, BOP, Et_3_N, THF, 3 h, rt, 78%; (**c**) HCl/dioxane, 1.5 h, rt, 78%.

Cyclohexyl analog **14** was prepared as shown in Fig. 7. (S)-3-methyl-1-(4-phenylpiperidin-1-yl)butan-2-amine **IV-1**, prepared according to previously reported methods^15^ was hydrogenated to afford the corresponding cyclohexyl intermediate **IV-2**. Routine BOP-mediated amidation of **IV-2** with carboxylic acid **I-1**, followed by HCl deprotection furnished **14**.

**Figure 8:**
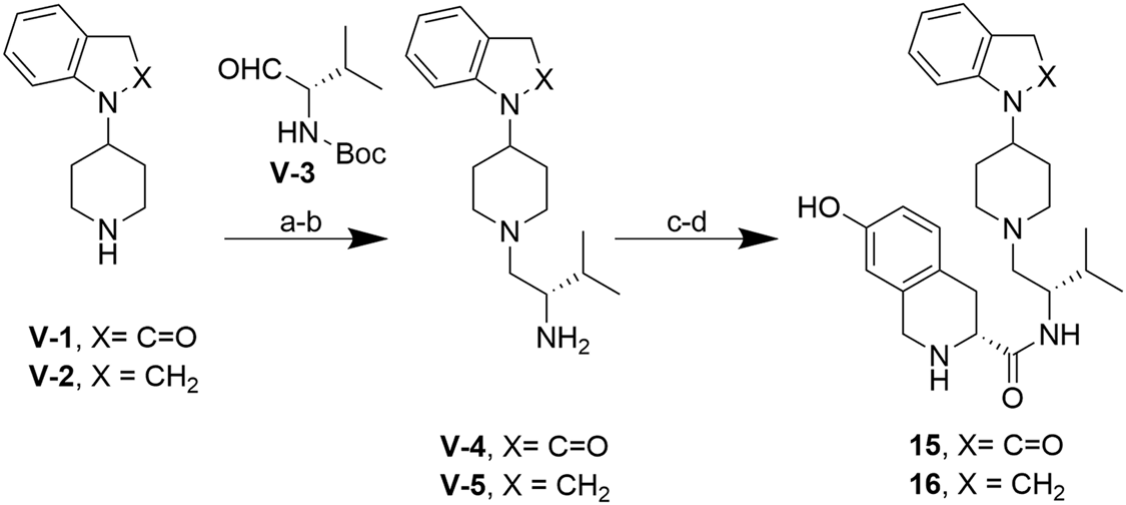
Synthesis of **15**–**16**. ***Reagents and conditions:*** (**a**) for **V-4**: HOAc, NaBH(OAc)_3_, 1,2 dichloroethane, 16 h, rt, 61%, for **V-5**: (i) HOAc, NaBH(OAc)_3_, Et_3_N, DCE, 16 h, rt, 62%, (ii) NaCNBH_3_, CF_3_CH_2_OH, 18 h, 50 °C, quantitative; (**b**) TFA, CH_2_Cl_2_, 2.5 h, rt, quantitative; (**c**) for **15**: **I**-**1**, propylphosphonic anhydride solution (T_3_P^®^), DiPEA, THF, 25 h, rt, 39%, for **16**: **I-1**, BOP, Et_3_N, THF, 23 h, rt, 29%; (**d**) HCl/dioxane, MeOH, rt, 1–2 h, 79–98%.

For the synthesis of indolinone analog **15** (Fig. 8), a reductive amination approach with 1-(piperidin-4-yl)-2,3-dihydro-1H-indol-2-one **V**-**1** ^31^ and commercially available Boc-L-valinal **V**-**3** was utilized, rather than an amidation-deprotection-reduction sequence previously used^15^, to avoid reduction of the indolinone lactam, decreasing the synthesis by one step and avoiding use of tedious borane. Trifluoroacetic acid deprotection of the reductive amination product then afforded intermediate **V-4**. Coupling of amine **V-4** with carboxylic acid **I**-**1** using propylphosphonic anhydride (T_3_P^®^), followed by HCl deprotection furnished **15**. Similar methodology was also used to access indoline **16**, starting from commercially available 1-(piperidin-4-yl)-2,3-dihydro-1H-indole **V-2**. Reductive amination with Boc-valinal **V-3** afforded the iminium intermediate, which was then reduced with NaCNBH_3_ in trifluoroethanol^32^. Deprotection with trifluoroacetic acid then afforded amine **V-5**. Subsequent BOP-mediated coupling of amine **V-5** with carboxylic acid **I-1**, followed by HCl deprotection gave **16**.

### *In vitro* pharmacological characterization

Compounds **2**–**16** were characterized *in vitro* for their binding affinities, intrinsic activity and antagonist potencies at the NOP, KOP, DOP and MOP receptors and compared to AT-076 (**1**) which was characterized in the same assays. Binding affinities of **1–16** at NOP, KOP, MOP and DOP were evaluated in radioligand competition experiments in membranes from CHO cells stably transfected with the respective human receptors, using the appropriate radioligands [^3^H]N/OFQ, [^3^H]U69,593, [^3^H]DAMGO and [^3^H]Cl-DPDPE, respectively, as described in the Methods^15,31,33,34^. Concentrations of the analogs showing 50% inhibition of radioligand binding (IC_50_) were determined from concentration-response curves and binding affinities reported as *K*_i_ (nM) (Table 1) calculated from the Cheng-Prusoff equation, as described in the Methods. The intrinsic activity of the compounds was determined by their ability to stimulate [^35^S]GTPγS binding to cell membranes in a six-point concentration curve up to 10 μM and compared to the standard agonists N/OFQ (NOP), DAMGO (MOP), U69,593 (KOP), and DPDPE (DOP), conducted as described in Methods^31,34–36^. None of the analogs tested had any intrinsic activity in the GTPγS assay at the four receptor subtypes.

**Table 1:** Binding affinity of **1** (AT-076) analogs at the four opioid receptors, determined in competition radioligand displacement assays in cloned human opioid receptor-transfected cells^a^.

Receptor Binding, *K*_*i*_ (nM)					
Cmpd	Structure	NOP	KOP	MOP	DOP
**1** (AT-076)		1.75 ± 0.74	1.14 ± 0.63	1.67 ± 0.6	19.6 ± 1.3
**2**		1890 ± 270	6.73 ± 1.42	81.7 ± 12.4	73.7 ± 57.9
**3**		5540 ± 1150	118 ± 29	1490 ± 220	1566 ± 1133
**4**		3260 ± 780	129 ± 60	180 ± 70	>10 K
**5**		7910 ± 1470	295 ± 65	4400 ± 2350	>10 K
**6**		>10 K	1518.4 ± 418.8	>10 K	>10 K
**7**		>10 K	125.34 ± 2.4	1442.1 ± 57.6	>10 K
**8**		61.32 ± 14.6	3.86 ± 0.09	2.93 ± 0.67	135.92 ± 59.7
**9**		>10 K	542.9 ± 62.2	972.91 ± 20.95	>10 K
**10**		30.72 ± 14.5	1.08 ± 0.01	0.68 ± 0.28	133.45 ± 24.9
**11**		202.14 ± 55.6	408.3 ± 30.8	284.0 ± 75.3	>10 K
**12**		6.04 ± 1.32	2.11 ± 0.52	5.67 ± 2.38	53.61 ± 7.12
**13**		43.32 ± 19.1	56.14 ± 5.38	408.33 ± 37.3	>10 K
**14**		84.4 ± 13.6	0.253 ± 0.015	2.79 ± 0.28	12.4 ± 8.0
**15**		91.19 ± 25.4	2.6 ± 1.13	19.08 ± 6.35	249.43 ± 31.0
**16**		566 ± 129	0.371 ± 0.096	7.68 ± 0.52	137.6 ± 45
N/OFQ		0.12 ± 0.01			
U69,593			1.05 ± 0.02		
DAMGO				2.96 ± 0.5	
DPDPE					1.11 ± 0.07
SB-612111		1.06 ± 0.52	541.26 ± 36.3	623.11 ± 156.3	2894.1 ± 532
JDTic		16.7 ± 0.76	0.43 ± 0.03	1.53 ± 0.19	10.6 ± 0.17

^a^ *K*_*i*_ values were determined by competitive displacement of the respective radioligands–[^3^H]N/OFQ–NOP, [^3^H]U69,593–KOP, [^3^H]DAMGO–MOP and [^3^H]DPDPE–DOP receptor. The *K*_*i*_ was calculated from the IC_50_ values determined from the binding curves, using the Cheng−Prusoff equation. Values are the Mean ± SEM of at least three independent experiments run in triplicate.

For compounds whose binding affinity *K*_i_ was < 50 nM, the antagonist potencies (pA_2_) were determined in the [^35^S]GTPγS functional assay using Schild analysis, where the shift in EC_50_ in the dose-response curve of the respective standard agonist is determined in the presence of at least 4 concentrations of the test antagonist. The pA_2_ values obtained in these analyses are shown in Table 2.

**Table 2:** Antagonist potencies (pA_2_) determined by Schild analysis in functional assays measuring inhibition of agonist-induced [^35^S]GTPγS binding at the four opioid receptors^¶^.

Antagonist Potency (pA_2_ ± SEM)				
	NOP	KOP	MOP	DOP
**1** (AT-076)	7.52	8.366	9.24	7.57
**2**	ND*	N/C^#^	ND	ND
**8**	ND	9.57 ± 0.18	8.09 ± 0.11	ND
**10**	6.88 ± 0.63	9.42 ± 0.13	7.81^§^	ND
**12**	8.30 ± 0.33	9.8 ± 0.8	9.16 ± 0.52	ND
**13**	N/C	N/C	ND	ND
**14**	ND	10.46 ± 0.0002	9.16 ± 0.11	7.3738 ± 0.31
**15**	ND	9.19 ± 0.16	7.98	ND
**16**	ND	10.56 ± 0.31	8.32	ND
SB-612111	9.28 ± 0.21	ND	ND	ND

^¶^pA_2_ values are given as mean ± SEM of at least two experiments performed in triplicate on two separate days.

*ND = antagonist potency was not determined for compounds whose binding affinity was >50 nM.

^#^N/C = compound showed a noncompetitive profile in Schild analysis.

^§^pA_2_ value from a single experiment done in triplicate.

## Results

To explore binding orientations of **1** ^15^ at the opioid receptors with an SAR analysis, we replaced the 7-hydroxy of the Tic moiety of **1** with positively charged aminergic substituents, as in analogs **3–7**, which, we hypothesized, may interact with anionic residues in the EL2 loops of the NOP and KOP receptors^37^. The uncharged nitrile analog **2** was also synthesized to explore the importance of the 7-OH in the Tic moiety. To our surprise, these modifications decreased binding affinity at all four receptors compared to that of the lead AT-076 (Table 1). The drop in affinity at NOP was particularly pronounced, over three orders of magnitude for **3**–**7** (see Table 1). At the KOP, MOP and DOP receptors, the effect of these modifications was less pronounced. The polar but uncharged nitrile analog **2** showed only a 6-fold drop in KOP affinity, but charged substituents as in analogs **3** and **4** caused a > 100-fold decrease in binding affinity at KOP, MOP and DOP receptors. Replacing the entire Tic-OH moiety with lysine (**6**) or arginine (**7**) significantly decreased affinity for all four receptors.

Structural modifications at the opposite end of the molecule, i.e. replacing the 3-hydroxyl group of the phenylpiperidine with an amino group (**10**), interestingly increased NOP binding affinity 2-fold compared to the uncharged cyano precursor (**8**), giving a *K*_*i*_ of 30.72 ± 14.5 nM. The arginine analog **12**, has even higher NOP affinity (*K*_*i*_ of 6.04 ± 1.32 nM), comparable to that of **1** at NOP. These modifications retained the high binding affinity at KOP and MOP but not the DOP receptors. Overall, the replacements of the 3-OH group were less detrimental to the affinity at NOP, and resulted in equi-potent binding affinity at KOP and MOP (analogs **8**, **10** and **12**). The importance of the 7-OH group in the Tic moiety was further confirmed with analogs **9**, **11** and **13**, because removal of this group significantly dropped affinity at all receptors, compared to **8**, **10** and **12** respectively.

To further investigate the binding orientation of AT-076 at the NOP receptor, the 3-hydroxyphenyl group of AT-076 was replaced with hydrophobic moieties such as a cyclohexyl ring (**14**), indolinone (**15**) and indoline (**16**). Interestingly, these analogs show a significant decrease in binding affinity at the NOP receptor.

However, at the KOP receptor, analogs **14** and **16** have sub-nanomolar affinity, being about 2–4-fold higher affinity than AT-076 itself. There was a modest decrease in affinity at MOP and DOP for these compounds compared to AT-076. With this enhancement of binding affinity at the KOP receptor, compounds **14** and **16** are selective KOP ligands, showing greater binding selectivity for KOP over MOP (11-fold for **14**, 20-fold for **16**), DOP (49-fold for **14**, 370-fold for **16**) and NOP (333-fold for **14**, >1000-fold for **16**) receptors, compared to the KOP antagonist JDTic, which shows only a 4-fold binding selectivity over MOP, 25-fold over DOP and 39-fold over NOP as determined in our experiments (Table 1).

Incorporation of a carbonyl group on **16** (to indolinone **15**) reduces KOP affinity by 10-fold, and shows decreased affinity at MOP and DOP compared to **16**.

Functional characterization of intrinsic (agonist) activity and antagonist potencies of the analogs was conducted using the [^35^S]GTPγS binding assay. As expected, none of the analogs had any agonist activity at any of the opioid receptors. On the other hand, several analogs that had nanomolar binding affinities for any of the opioid receptors also showed significant antagonist potencies at that receptor, reported as pA_2_ values shown in Table 2. Notably, compound **12**, which has single digit nanomolar binding affinity at NOP, KOP and MOP (Table 1) also has high antagonist potency at these receptors (pA_2_ values 8.3, 9.8 and 9.2 respectively, Table 2). The subnanomolar binding affinity and high selectivity for KOP observed with analogs **14** and **16** also translate to high antagonist potencies at KOP for these compounds (pA_2_ −10.5 and −10.6, Table 2). Compounds **14** and **16** are therefore selective and potent KOP antagonists.

## Discussion

The nanomolar binding affinity of AT-076 (**1)** at all four opioid receptors suggests that it has a chemotype that binds in the opioid binding pocket of all four opioid receptors, NOP, MOP, KOP and DOP^15^. **1** may possess a common opioid pharmacophore and can be used as a tool compound to probe ligand recognition features at all opioid receptors. Such information is useful for the design of multifunctional or selective opioid ligands, as needed, based on this scaffold. To aid such studies, we continued our SAR studies of **1** and investigated several chemical structure modifications, designed to inform the SAR at all four opioid receptors.

We previously reported the results of docking compound **1** in the NOP receptor crystal structure bound to antagonist C-24 (PDB No: 4EA3)^15^. **1** was found to bind in an extended conformation to NOP^15^, similar to the co-crystallized NOP antagonist C-24^18^. The piperidine nitrogen of **1** formed a salt bridge with the conserved Asp130^3.32^, a key interaction for ligands at all four opioid receptors. Similar to the benzofuran moiety of C-24, the 3-hydroxyphenyl moiety of **1** at the 4-position of the central piperidine ring, was oriented toward the intracellular end of the NOP binding site in a lipophilic pocket, comprised of Tyr 131^3.33^, Met 134^3.36^ and Trp276^6.48^. The opposite end of the molecule, i.e. the Tic-OH moiety, of AT-076 in this docked pose was oriented towards the extracellular end of the binding cavity towards the EL2 loop, enriched with anionic residues such as Glu196, Glu197 and Glu199 in NOP^3,18,37^. However, the SAR of analogs **2**–**7** showed a large drop in NOP receptor binding affinity when the 7-OH group of the Tic-OH moiety was replaced with positively charged groups designed to interact with these anionic residues of the EL2 loop (Table 1 and Fig. 2). On the other hand, similar modifications of the 3-OH of the phenylpiperidine moiety retained NOP binding affinity similar to that of AT-076 (compound **12**). SAR of analogs **2–7** suggests that AT-076 may not bind in the same orientation as the co-crystallized NOP antagonist C-24 as previously suggested by our docking results^15^. On the other hand, the high affinity of compounds **10** and **12** at NOP suggests that the positively charged moiet(ies) replacing the 3-OH of the phenylpiperidine instead, may likely contribute to the high affinity by interacting with the negatively charged residues near the extracellular end and EL2 loop of the NOP binding pocket. This SAR supports a reversed binding mode than previously proposed, such that the 3-hydroxyphenyl on the piperidine ring is oriented towards the extracellular end of the NOP binding pocket, rather than towards the intracellular end, as previously found in the docked pose of **1** ^15^. Such an orientation would place the 7-hydroxy-4-(3-hydroxyphenyl)-1-piperidinyl]methyl}-2-methylpropyl)-1,2,3,4-tetrahydro-3-isoquinolinecarboxamide in the hydrophobic pocket, lined with residues conserved among the four opioid subtypes such as Met134^3.36^. SAR showing poor NOP binding affinities for analogs **14** and **16**, bearing hydrophobic replacements of the 3-hydroxyphenyl moiety, further suggests that these groups at the C-4 position of the central piperidine ring are likely oriented towards the polar extracellular end of the NOP binding pocket, supporting a flipped orientation of AT-076 analogs compared to co-crystallized antagonist C-24 at the NOP receptor.

Our SAR results suggest that NOP ligands of a chemotype different from the co-crystallized ligand C-24 may possibly bind in a different orientation than the co-crystallized ligand. Indeed, docking studies of other piperidine-based NOP antagonists J-113397 and its analog Trap-101 in the NOP crystal structure conducted by Miller *et al*.^24^ showed that these antagonists favored the ‘flipped’ orientation, in which the piperidine C-4 heterocycle is oriented towards the extracellular end of the binding pocket, whereas the piperidine N-1 cyclooctyl substituent is buried in the intracellular hydrophobic pocket. SAR studies such as reported here are therefore useful for investigating possible binding orientations of ligand chemotypes different than the co-crystallized ligands in the opioid receptor crystal structures.

**Figure 9:**
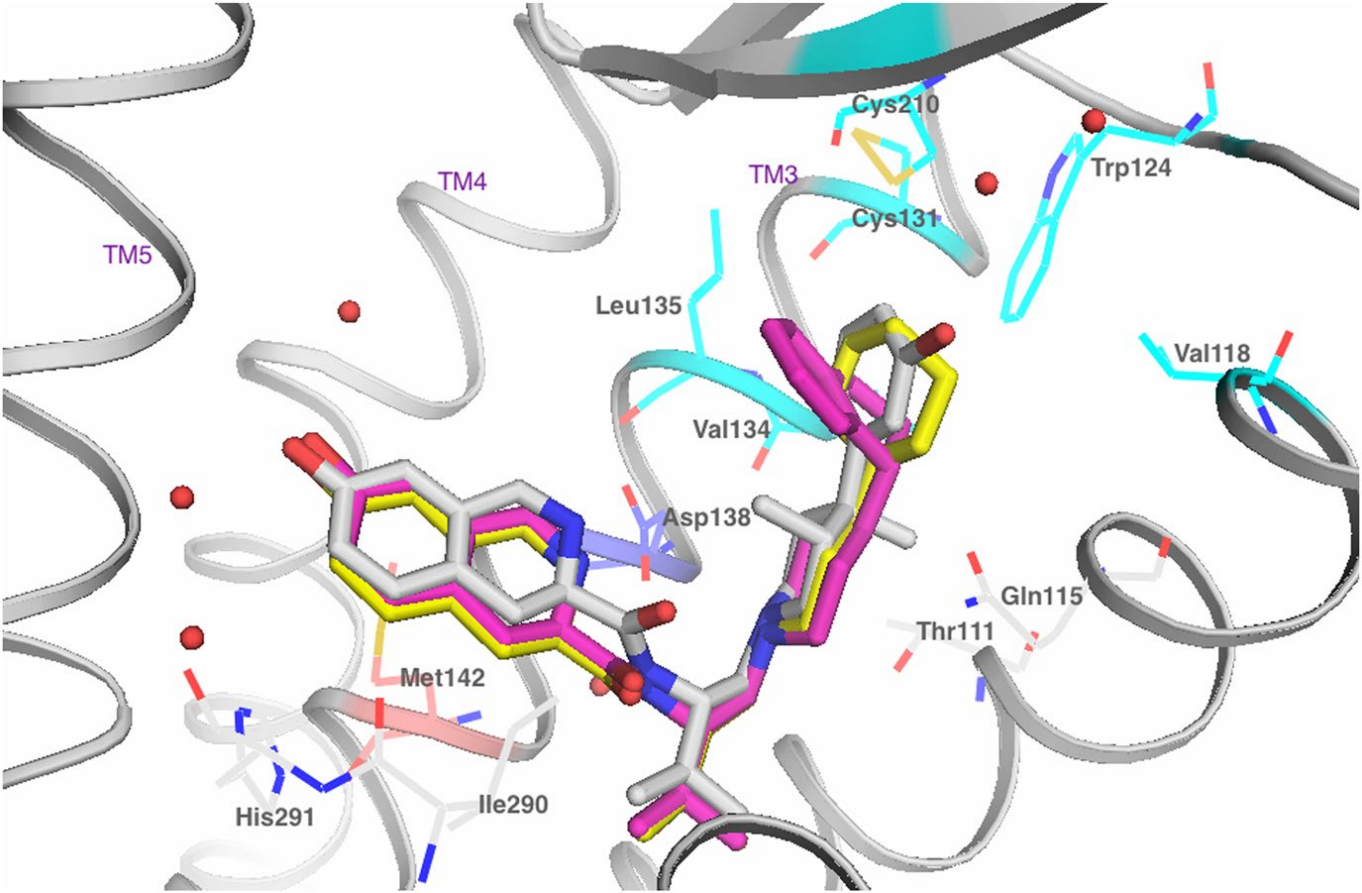
Docking of analogs **14** (yellow) and **16** (magenta) in the KOP crystal structure 4DJH. Co-crystallized ligand JDTic shown in white. The TM helices 3, 4 and 5 are labeled. TM6 is not shown for clarity. The amino acid side chains within 4 Å of the ligands are depicted. The ionic interaction between Asp138^3.32^ and the charged piperidine nitrogen of the antagonists is shown as a red dashed line. Both **14** and **16** have subnanomolar affinity and high selectivity for KOP. Their 4-piperidine substituents (cyclohexyl in **14** and indolinyl in **16**) appear to interact with hydrophobic residues Val118^2.63^, Cys131^3.25^, Val134^3.28^ and Leu135^3.29^ in the KOP structure 4DJH.

At the KOP receptor, the high KOP selectivity and antagonist potency of analogs **14** and **16** suggests that these compounds likely bind to the KOP receptor in an orientation similar to that of JDTic in the KOP receptor crystal structure^17^. The 3-hydroxyphenyl ring of JDTic is situated in a pocket comprised of Val118^2.63^, Cys131^3.25^, Val134^3.28^ and Leu135^3.29^ (See Fig. 9). These residues could likely provide strong hydrophobic interactions for the cyclohexyl and indoline group replacements in **14** and **16**, respectively, which may explain their high binding affinity. Docking the selective KOP antagonists **14** and **16** in the KOP crystal structure 4DJH^17^ confirmed this binding orientation similar to that of JDTic, in which the cyclohexyl and indoline groups of in **14** and **16** occupy the same pocket as the 3-hydroxyphenyl ring of JDTic, as shown in Fig. 9.

## Conclusions

In summary, this SAR study of **1** reveals several interesting trends–(i) **1** represents an universal opioid antagonist chemotype that is not a morphinan scaffold. (ii) The 7-hydroxy-1,2,3,4-tetrahydro-3-isoquinolinecarboxamide appears to be important pharmacophore for binding at all four opioid receptors since modifications in this moiety (**2–7**, **9**, **11** and **13**) causes significant loss of affinity at all four receptors. (iii) Substituents at the 4-position of the piperidinyl ring may be used to modulate affinity and selectivity, particularly for the KOP receptor. Such modifications resulted in the discovery of a selective KOP antagonist **16** from a pan antagonist lead compound **1**. (iv) The SAR for **1** and its analogs at the NOP receptor highlights the limitations of docking using the X-ray crystal structures as a *single* tool for rational drug design. Rather, a combination of experimental SAR and docking allows for an accurate understanding of ligand recognition of structurally diverse ligands at the opioid receptors.

## Methods

Thin layer chromatography was performed on Analtech silica gel GF 250 micron TLC plates. The plates were visualized with a 254 nM UV light and staining with iodine. Flash chromatography was carried out on F60 silica gel, 230–400 mesh, 60 Å (Silicycle SiliaFlash). NMR was recorded on a Varian Mercury Plus NMR (300 MHz), Varian Mercury 300 (300 MHz), or Varian 400 (400 MHz) using CDCl_3_ or MeOD-*d*_4_. Mass spectra were obtained on a LCQ Fleet Ion Trap LC/MS^n^, a micromass ZMD 1000 or PE Sciex API 150EX LC/MS using electrospray ionization (ESI) or atmospheric pressure chemical ionization (APCI) mode. Elemental analyses were performed by Atlantic Microlabs, Norcross, GA. HRMS analyses were performed by the Mass Spectrometry Service Laboratory, University of Minnesota Department of Chemistry, Minneapolis, MN on a Bruker BioTOF II HRMS using ESI mode. For final compounds **6** and **7**, HPLC analysis was performed on a reverse phase Varian C18 column (2.0 × 50 mm), using a binary gradient of 95:5 solvent A (H_2_O + 0.1% formic acid): solvent B (ACN) → 0:100 for 15 minutes, at a flow rate of 200 μL/min. Eluted peaks were monitored at 254 nm with a Finnigan Surveyor PDA Plus detector. For all remaining final compounds, HPLC analysis was performed on a reverse phase Agilent Zorbax SB-Phenyl column (5 µm, 2.1 × 150 mm), using a binary gradient of 95:5 solvent A (95/5 H_2_O/ACN + 0.1% formic acid):solvent B (5/95 H_2_O/ACN + 0.1% formic acid) → 0:100 for 10 minutes, at a flow rate of 0.4 mL/min. Eluted peaks were monitored at 254 nm with a Shimadzu SPD-10AVP UV-Vis detector. All final compounds tested were confirmed to be of > 95% purity by the HPLC method described above.

### General Procedure 1: Conversion of phenol to aryl triflate

To a stirred solution of the appropriate phenol intermediate (1.00 equiv) in THF (0.10 M) was added N-phenyl-bis(trifluoromethanesulfonimide) (3.00 equiv) and K_2_CO_3_ (8.00 equiv) under Ar(g), and the mixture stirred at room temperature for 18–26 h. The reaction was diluted with H_2_O and CH_2_Cl_2_.The layers were separated, and the aqueous solution was extracted 2X with CH_2_Cl_2_. The combined organic layers were washed with satd. NaCl(aq), dried over Na_2_SO_4_, filtered and concentrated. The crude residue was purified via flash chromatography (for **I-2**: Hexanes/EtOAc 95/5 → 75/25, for **III-3a**: Hexanes/EtOAc 90/10 → 40/60, for **III-3b**: Hexanes/EtOAc 90/10 → 30/70) to afford the desired material.

### General Procedure 2: Pd-catalyzed cyanation of aryl triflates

To a stirred solution of the appropriate triflate intermediate (1.00 equiv) dissolved in DMF (0.10–0.13 M) was added ZnCN_2_ (1.00–1.70 equiv), and Ar(g) was bubbled through the mixture for 15 min. Pd(PPh_3_)_4_ (0.11–0.2 equiv) was added, and the mixture was heated at 80–100 °C for 1–16 h. The reaction was diluted with H_2_O and EtOAc. The layers were separated, and the aqueous solution was extracted 2X with EtOAc. The combined organic layers were washed with satd. NaCl(aq), dried over Na_2_SO_4_, filtered and concentrated. The crude residue was purified via flash chromatography (for **I-3**: Hexanes/EtOAc 95/5 → 75/25, for **III-4a**: Hexanes/EtOAc/NH_4_OH(aq) 95/5/1 → 50/50/1, for **III-4b**: Hexanes/EtOAc/NH_4_OH 90/10/1 → 40/60/1) to afford the desired material.

### General Procedure 3: Amidation

**Method A** **:** To a stirred solution of amine (1.00 equiv) in THF (0.1 M) was added the appropriate carboxylic acid (1.20 equiv), BOP (1.20 equiv) and Et_3_N (5.00 equiv), and the reaction was stirred at room temperature for 2–23 h. The reaction was diluted with EtOAc and satd. NaHCO_3_(aq). The layers were separated, and the aqueous solution was extracted 2X with EtOAc. The combined organic layers were washed with satd. NaCl(aq), dried over Na_2_SO_4_, filtered and concentrated. The crude residue was purified via flash chromatography (for **I-5**: Hexane/EtOAc/NH_4_OH 50/50/0 → 0/100/1, for **II-1**: CH_2_Cl_2_/iPrOH 100/0 → 95/5, for **II-2**: Hexane/EtOAc/NH_4_OH 79.5/20/0.5 → 49.75/49.75/0.5, for **III-2a**: Hexanes/CH_2_Cl_2_/EtOAc 50/50/0 → 0/0/100 then EtOAc/MeOH 99/1, for **14**: Hexane/EtOAc/NH_4_OH 90/10/0 → 40/60/1, for **16**: Hexane/EtOAc/iPrOH/NH_4_OH 50/50/0/0 → 25/25/50/1) to afford the desired material. **Method B**: To a stirred solution of Boc-Arg(Boc)_2_-OH (1.20 equiv) in CH_2_Cl_2_ or DMF (0.04 M) was added HOBt (1.15–1.20 equiv), EDCI (1.2 equiv) and Et_3_N (1.40–3.40 equiv), and the reaction was stirred at room temperature for 2 h. To this mixture was added a solution of amine (1.00 equiv) in CH_2_Cl_2_ or DMF, and the reaction was stirred at room temperature for 24 h. The reaction was diluted with CH_2_Cl_2_ and satd. NaHCO_3_(aq). The layers were separated, and the aqueous solution was extracted 2X with CH_2_Cl_2_. The combined organic layers were washed with satd. NaCl(aq), dried over Na_2_SO_4_, filtered and concentrated. The crude residue was purified via flash chromatography (for **III-6a**: Hexane/EtOAc/NH_4_OH 95/5/0 → 30/70/1, for **III-6b**: Hexane/EtOAc 90/10 → 40/60) to afford the desired material.

### General Procedure 4: Boc deprotection

**Method A** **:** To a solution of Boc-protected intermediate (1.00 equiv) in CH_2_Cl_2_ was added TFA (1:1 or 1:2 v:v, 0.02–0.05 M), and the reaction was stirred at room temperature for 1–5.5 h. **Method B**: To a solution of Boc-protected intermediate (1.00 equiv) in CH_2_Cl_2_ (0.1 M) was added TFA (8–10 equiv), and the reaction was stirred at room temperature for 2.5–4 h. **Method C**: To a solution of Boc-protected intermediate (1.00 equiv) in MeOH (0.12–0.21 M) was added 4 M HCl/dioxane (55–165 equiv), and the reaction was stirred at room temperature for 1–11 h. The reaction was then concentrated to a solid.

### General Procedure 5: Nitrile reduction

Cyano intermediate (1.00 equiv) was dissolved in 7 N NH_3_/MeOH (0.04–0.31 M), and Raney Nickel (75–100 wt % of substrate) was added. The mixture was hydrogenated at 4–4.8 atm H_2_(g) for 5.5–21 h at room temperature, then filtered through a pad of Celite and concentrated. The residue was purified by flash chromatography (for **III-5a**: CH_2_Cl_2_/MeOH/NH_4_OH(aq) 100/0/0 → 90/10/0.5, for **III-5b**: CH_2_Cl_2_/MeOH/NH_4_OH(aq) 100/0/0 → 20/80/1, for **10**: CH_2_Cl_2_/MeOH/NH_4_OH(aq) 100/0 → 93/6/1) to afford the desired material.

### General Procedure 6: TBDMS-deprotection

To a mixture of TBDMS protected intermediate (1.00 equiv) in THF (ah) (0.06-M) was added TBAF/THF (0.88 equiv), and the reaction was stirred at room temperature under Ar(g) for 1–2 h. The reaction was diluted with EtOAc and NaCl(aq). The layers were separated, and the aqueous solution was extracted 2X with EtOAc. The combined organic layers were washed with satd. NaCl(aq), dried over Na_2_SO_4_, filtered and concentrated. The residue was purified by flash chromatography (for **8**: CH_2_Cl_2_/iPrOH 100/0 → 93/7; for **III-7a**: Hexane/EtOAc/NH_4_OH 80/20/0 → 70/30/1) as the eluent to afford the desired material.

### 2-(tert-butyl) 3-methyl (R)-7-(((trifluoromethyl)sulfonyl)oxy)-3,4-dihydroisoquinoline-2,3(1 H)-dicarboxylate (I-2)

To a solution of (3 R)-2-(tert-Butoxycarbonyl)-7-hydroxy-1,2,3,4-tetrahydroisoquinoli ne-3-carboxylic acid **I-1** (0.97 g, 3.29 mmol) in acetone (33.0 mL) was added LiOH (138 mg, 3.29 mmol, 1.00 equiv) and dimethyl sulfate (313 μL, 3.29 mmol, 1.00 equiv), and the reaction was gently refluxed at 65 °C for 5 h. The reaction was cooled in an ice bath for 20 min, then filtered and concentrated to afford 1.50 g of 2-(tert-butyl) 3-methyl (R)-7-hydroxy-3,4-dihydroisoquinoline-2,3(1 H)-dicarboxylate in quantitative yield. ^1^H NMR (300 MHz, CDCl_3_) *δ* 7.00 (1 H, d, *J* = 9 Hz), 6.59–6.70 (2 H, m), 5.10–5.12 (1 H, m), 4.60–4.76 (1 H, m), 4.39–4.49 (1 H, m), 4.24 (1 H, br s), 3.63 (3 H, app d, *J* = 9 Hz), 3.06–3.19 (2 H, m), 1.49 (9 H, app d, *J* = 18 Hz). MS(ESI) *m/z* 208.3 (M + H-Boc)^+^. The title material was prepared according to General Procedure 1 using the methyl ester intermediate (1.45 g, 4.72 mmol) to afford 1.21 g of the title material in 83% yield. ^1^H NMR (300 MHz, CDCl_3_) *δ* 7.23 (1 H, d, *J* = 9 Hz), 7.04–7.11 (2 H, m), 5.19–5.21 (1 H, m), 4.72–4.85 (1 H, m), 4.50 (1 H, t, *J* = 15 Hz), 3.64 (3 H, app d, *J* = 6 Hz), 3.11–3.32 (2 H, m), 1.46–1.55 (9 H, m).

### (R)-2-(tert-butoxycarbonyl)-7-cyano-1,2,3,4-tetrahydroisoquinoline-3-carboxylic acid (**I-3**)

Intermediate **I-2** (3.94 g, 8.97 mmol) was subjected to conditions described in General Procedure 2 to afford 2.44 g of 2-(tert-butyl) 3-methyl (R)-7-cyano-3,4-dihydroisoquinoline-2,3(1 H)-dicarboxylate in 86% yield. ^1^H NMR (400 MHz, CDCl_3_) *δ* 7.41–7.47 (2 H, m), 7.24–7.26 (1 H, m), 5.19–5.20 (1 H, m), 4.74 (1 H, dd, *J* = 16, 4 Hz), 4.49 (1 H, t, *J* = 20 Hz), 3.63 (3 H, app d, *J* = 4 Hz), 3.15–3.33 (2 H, m, *J* = 4 Hz), 1.49 (9 H, app d, *J* = 28 Hz). To a solution of the cyano intermediate (1.12 g, 3.54 mmol) in THF (0.1 M) was added 1 M LiOH (aq) (3.00 equiv), and the reaction was stirred at room temperature for 1 h. The reaction was transferred to an oversized beaker, diluted with EtOAc and H_2_O and cooled in an ice bath. The pH of the aqueous layer was adjusted to pH = 4 by addition of HOAc. The layers were separated, and the aqueous solution was extracted 3X with EtOAc. The combined organic layers were dried over Na_2_SO_4_, filtered and concentrated to afford 1.18 g of the crude title material in quantitative yield. ^1^H NMR (400 MHz, CDCl_3_) *δ* 7.42–7.47 (2 H, m), 7.25 (1 H, s), 5.12 (1 H, s), 4.66–4.73 (1 H, m), 4.49 (1 H, d, *J* = 20 Hz), 3.20–3.32 (2 H, m), 1.46 (9 H, app d, *J* = 32 Hz).

### tert-butyl (R)-7-cyano-3-(((S)-1-(4-(3-hydroxyphenyl)piperidin-1-yl)-3-methylbutan-2-yl)carbamoyl)-3,4-dihydroisoquinoline-2(1 H)-carboxylate (**I-5**)

Prepared according to General Procedure 3 Method A using (S)-3-(1-(2-amino-3-methylbutyl)piperidin-4-yl)phenol (**I-4**)^15^ (645 mg, 1.00 mmol), intermediate **I-3** (780 mg, 2.58 mmol), BOP (2.61 g, 2.4 mmol) and Et_3_N (4.11 mL, 29.5 mmol) to afford 995 mg of the title material in 74% yield. ^1^H NMR (300 MHz, CDCl_3_) *δ* 7.46 (2 H, t, *J* = 9 Hz), 7.33 (1 H, d, *J* = 6 Hz), 7.18 (1 H, t, *J* = 9 Hz), 6.69–6.73 (3 H, m), 6.01 (1 H, br s), 4.56–5.02 (2 H, m), 3.85 (1 H, br s), 3.49 (1 H, d, *J* = 15 Hz), 3.05 (1 H, dd, *J* = 15, 6 Hz), 2.81 (2 H, br s), 2.13–2.41 (3 H, m), 1.66–1.83 (5 H, m), 1.52 (9 H, s), 1.31–1.47 (2 H, br s), 0.87 (6 H, app dd, *J* = 12, 9 Hz). MS(ESI) *m/z* 547.4 (M + H)^+^.

### (R)-7-cyano-N-((S)-1-(4-(3-hydroxyphenyl)piperidin-1-yl)-3-methylbutan-2-yl)-1,2,3,4-tetrahydroisoquinoline-3-carboxamide (**2**)

Prepared according to General Procedure 4 Method C from intermediate **I-5** (115 mg, 0.21 mmol). The crude was diluted with CH_2_Cl_2_ and satd. NaHCO_3_(aq). Solid NaHCO_3_ was added. The layers were separated, and the aqueous solution was extracted 2X with CH_2_Cl_2_. The combined organic layers were washed with satd. NaCl(aq), dried over Na_2_SO_4_, filtered and concentrated. The crude residue was purified via flash chromatography using CH_2_Cl_2_/iPrOH/NH_4_OH 100/0/0 → 94/6/0.5 to afford 38 mg of the title material in 40% yield, which was converted to the HCl salt by addition of 4 M HCl/dioxane. ^1^H NMR (300 MHz, CDCl_3_) *δ* 7.41 (1 H, d, *J* = 6 Hz), 7.31 (1 H, s), 7.05–7.22 (2 H, m), 6.61–6.64 (3 H, m), 3.91–4.09 (3 H, m), 3.58–3.63 (1 H, m), 3.18 (2 H, dd, *J* = 18, 6 Hz), 2.93–3.02 (2 H, m), 2.17–2.55 (4 H, m), 1.56–1.97 (6 H, m), 0.94 (6 H, app t, *J* = 9 Hz). MS(ESI) *m/z* 447.4 (M + H)^+^. Anal. Calcd. for C_27_H_34_N_4_O_2_^.^2.00 HCl^.^2.00 H_2_O^.^0.4 Dioxane^.^0.7 MeOH: C, 57.39; H, 7.56; N, 9.14; found: C, 57.05; H, 7.24; N, 8.88.

### tert-butyl (R)-7-(aminomethyl)-3-(((S)-1-(4-(3-hydroxyphenyl)piperidin-1-yl)-3-methylbutan-2-yl)carbamoyl)-3,4-dihydroisoquinoline-2(1 H)-carboxylate (**I-6**)

Prepared according to General Procedure 5 using intermediate **I-5** (1.06 g, 1.94 mmol) to afford 1.06 g of the crude title material in 99% yield. ^1^H NMR (300 MHz, MeOD-*d*_4_) *δ* 7.15 (3 H, br s), 7.07 (1 H, t, *J* = 9 Hz), 6.58–6.66 (3 H, m), 4.78 (1 H, br s), 4.62 (2 H, br s), 3.81–3.87 (1 H, m), 3.73 (2 H, s), 3.14–3.20 (2 H, m), 2.81 (2 H, d, *J* = 9 Hz), 2.32–2.40 (3 H, m), 1.93–1.98 (2 H, m), 1.58–1.73 (5 H, m), 1.50 (9 H, s), 0.82 (6 H, app s). MS(ESI) *m/z* 551.6 (M + H)^+^.

### (R)-7-(aminomethyl)-N-((S)-1-(4-(3-hydroxyphenyl)piperidin-1-yl)-3-methylbutan-2-yl)-1,2,3,4-tetrahydroisoquinoline-3-carboxamide (**3**)

Prepared according to General Procedure 4 Method C from pure intermediate **I-6** (66 mg, 0.12 mmol) to afford 82 mg of the title material as a 3 HCl salt in quantitative yield. ^1^H NMR (300 MHz, MeOD-*d*_4_) *δ* 7.37–7.42 (3 H, m), 7.12 (1 H, t, *J* = 6 Hz), 6.64–6.78 (3 H, m), 4.51 (2 H, s), 4.43 (1 H, dd, *J* = 12, 6 Hz), 4.33 (1 H, br s), 4.10–4.13 (3 H, br s), 3.56–3.61 (1 H, m), 3.37–3.48 (3 H, m), 3.19–3.27 (2 H, m), 3.07 (1 H, t, *J* = 12 Hz), 2.78–2.86 (1 H, m), 2.50–2.63 (1 H, m), 2.20–2.34 (1 H, m), 1.86–2.05 (3 H, m), 1.02–1.05 (6 H, m). MS(ESI) *m/z* 451.4 (M + H)^+^. Anal. Calcd. for C_27_H_38_N_4_O_2_^.^3.00 HCl^.^2.00 H_2_O^.^1.20 Dioxane: C, 54.43; H, 7.84; N, 7.98; found: C, 54.18; H, 7.67; N, 7.83.

### tert-butyl (R)-7-(((E)-2,3-bis(tert-butoxycarbonyl)guanidino)methyl)-3-(((S)-1-(4-(3-hydroxyphenyl)piperidin-1-yl)-3-methylbutan-2-yl)carbamoyl)-3,4-dihydroisoquinoline-2(1 H)-carboxylate (**I-7**)

To a solution of crude intermediate **I-6** (250 mg, 0.45 mmol, 1.00 equiv) in THF (4.5 mL) was added Et_3_N (158 μL, 1.13 mmol, 2.50 equiv), 1,3-bis (*tert*-butoxycarbonyl)-2-methyl-2-thiopseudourea (158 mg, 0.54 mmol, 1.20 equiv) and HgCl_2_ (148 mg, 0.54 mmol, 1.20 equiv), and the reaction was stirred at room temperature for 2.5 h. The reaction was filtered through a pad of Celite, and concentrated. The crude residue was purified via flash chromatography using CH_2_Cl_2_/MeOH/NH_4_OH 100/0/0 → 98/2/0.5 to afford 129 mg of the title material in 36% yield. MS(ESI) *m/z* 793.7 (M + H)^+^.

### (R)-7-(guanidinomethyl)-N-((S)-1-(4-(3-hydroxyphenyl)piperidin-1-yl)-3-methylbutan-2-yl)-1,2,3,4-tetrahydroisoquinoline-3-carboxamide (**4**)

Prepared according to General Procedure 4 Method C from intermediate **I-7** (121 mg, 0.15 mmol) to afford 113 mg of the title material as a 3 HCl salt in quantitative yield. ^1^H NMR (300 MHz, MeOD-*d*_4_) *δ* 7.25–7.36 (3 H, m), 7.12 (1 H, t, *J* = 6 Hz), 6.73–6.78 (2 H, m), 6.64–6.67 (1 H, m), 4.49 (2 H, s), 4.39–4.42 (3 H, br s), 4.30–4.36 (1 H, m), 4.11 (1 H, d, *J* = 12 Hz), 3.56–3.65 (1 H, m), 3.35–3.45 (2 H, m), 3.18–3.27 (3 H, m), 3.02–3.11 (1 H, m), 2.77–2.85 (1 H, m), 2.50–2.63 (1 H, m), 2.19–2.34 (1 H, m), 1.86–2.05 (3 H, m), 1.01–1.05 (6 H, m). LCMS R_T_ = 1.20 min; *m/z* (M + H)^+^  = 493.4. HRMS (ESI) Calcd for C_28_H_41_N_6_O_2_ (M + H)^+^ 493.3285; found 493.3284.

### tert-butyl (R)-3-(((S)-1-(4-(3-hydroxyphenyl)piperidin-1-yl)-3-methylbutan-2-yl)carbamoyl)-7-(methylsulfonamidomethyl)-3,4-dihydroisoquinoline-2(1 H)-carboxylate (**I-8**)

To a solution of crude intermediate **I-6** (250 mg, 0.45 mmol, 1.00 equiv) in CH_2_Cl_2_ (0.1 M) was added pyridine (2.00 equiv) under Ar(g), and the reaction was cooled for 10 min. in an ice bath. Methanesulfonyl chloride (1.00 equiv) was added to the mixture, and the reaction was stirred at room temperature for 16 h. The reaction was diluted with CH_2_Cl_2_ and satd. NaHCO_3_(aq). The layers were separated, and the aqueous solution was extracted 2X with CH_2_Cl_2_. The combined organic layers were washed with satd. NaCl(aq), dried over Na_2_SO_4_, filtered and concentrated. The crude residue was purified via flash chromatography using CH_2_Cl_2_/iPrOH/NH_4_OH 100/0/0 → 94/6/0.5 to afford 142 mg of the title material in 50% yield. ^1^H NMR (300 MHz, CDCl_3_) *δ* 7.09–7.18 (4 H, m), 6.69–6.72 (3 H, m), 6.07 (1 H, br s), 4.53–5.01 (2 H, m), 4.09–4.16 (2 H, m), 3.89 (1 H, br s), 3.42 (2 H, dd, *J* = 18, 3 Hz), 2.97–3.02 (1 H, m), 2.85 (3 H, s), 2.60–2.81 (2 H, m), 2.09–2.39 (3 H, m), 1.78 (3 H, br s), 1.52–1.62 (13 H, m), 0.88 (6 H, app dd, *J* = 9, 6 Hz). MS(ESI) *m/z* 629.6 (M + H)^+^.

### (R)-N-((S)-1-(4-(3-hydroxyphenyl)piperidin-1-yl)-3-methylbutan-2-yl)-7-(methylsulfonamidomethyl)-1,2,3,4-tetrahydroisoquinoline-3-carboxamide (**5**)

Prepared according to General Procedure 4 Method C from intermediate **I-8** (82 mg, 0.13 mmol) to afford 89 mg of the title material as a 2 HCl salt in quantitative yield. ^1^H NMR (300 MHz, MeOD-*d*_4_) *δ* 7.29–7.36 (3 H, m), 7.12 (1 H, t, *J* = 6 Hz), 6.73–6.78 (2 H, m), 6.66 (1 H, d, *J* = 9 Hz), 4.47 (2 H, s), 4.30–4.42 (2 H, m), 4.24 (2 H, s), 4.12 (1 H, d, *J* = 9 Hz), 3.59 (1 H, br s), 3.36–3.43 (2 H, m), 3.17–3.26 (3 H, m), 3.06 (1 H, t, *J* = 12 Hz), 2.89 (3 H, s), 2.76–2.84 (1 H, m), 2.48–2.61 (1 H, m), 2.19–2.33 (1 H, m), 1.86–2.05 (3 H, m), 1.03 (6 H, app t, *J* = 6 Hz). MS(ESI) *m/z* 529.3 (M + H)^+^. Anal. Calcd. for C_28_H_40_N_4_O_4_S^.^2.00 HCl^.^2.00 H_2_O^.^0.60 Dioxane: C, 52.88; H, 7.42; N, 8.11; found: C, 52.57; H, 7.10; N, 7.88.

### dibenzyl ((S)-6-(((S)-1-(4-(3-hydroxyphenyl)piperidin-1-yl)-3-methylbutan-2-yl)amino)-6-oxohexane-1,5-diyl)dicarbamate (**II-1**)

Prepared according to General Procedure 3 Method A using (S)-3-(1-(2-amino-3-methylbutyl)piperidin-4-yl)phenol (**I-4**)^15^ (100 mg, 0.38 mmol) and N2,N6-Bis(benzyloxycarbonyl)-L-lysine (190 mg, 0.46 mmol) to afford 160 mg of the title material in 64% yield. ^1^H NMR (300 MHz, CDCl_3_) *δ* 7.27–7.40 (10 H, m), 7.14 (1 H, t, *J* = 5.7 Hz), 6.83 (1 H, s), 6.66–6.74 (2 H, m), 6.20 (1 H, s), 5.26 (1 H, d, *J* = 9 Hz), 5.06–5.09 (2 H, m), 4.93 (1 H, s), 4.54 (1 H, s), 4.02–4.07 (2 H, m), 3.97 (1 H, s), 3.54 (1 H, s), 2.95–3.23 (3 H, m), 2.70–2.73 (2 H, m), 1.43–2.40 (13 H, m), 1.22 (4 H, d, *J* = 4.5 Hz), 0.89–0.96 (6 H, m). MS(ESI) *m/z* 659.64 (M + H)^+^.

### (S)-2,6-diamino-N-((S)-1-(4-(3-hydroxyphenyl)piperidin-1-yl)-3-methylbutan-2-yl)hexanamide (**6**)

To a solution of intermediate **II-1** (140 mg, 0.21 mmol) in THF (3.54 mL) was added Pd/C, 10% (35 mg), and the reaction was stirred at room temperature for 6 h under an atmosphere of H_2_(g). The solution was filtered through a pad of Celite and concentrated to afford 83 mg of the title material in quantitative yield, which was converted to the HCl salt by addition of 2 M HCl/ether. ^1^H NMR (300 MHz, MeOD-*d*_4_) *δ* 7.08 (1 H, t, *J* = 6 Hz), 6.58–6.69 (3 H, m), 3.96–4.02 (1 H, m), 3.37 (1 H, t, *J* = 6 Hz), 3.15 (1 H, d, *J* = 12 Hz), 2.88–3.01 (3 H, m), 2.40–2.52 (3 H, m), 2.26 (1 H, td, *J* = 12, 3 Hz), 2.07 (1 H, td, *J* = 12, 3 Hz), 1.48–1.88 (11 H, m), 0.94 (6 H, app t, *J* = 6 Hz). HRMS (ESI) Calcd for C_22_H_39_N_4_O_2_ (M + H)^+^ 391.3068; found 391.3050. LCMS R_T_ = 0.78 min; *m/z* (M + H)^+^  = 391.38.

### tert-butyl N-[(1E)-{[(4 S)-4-{[(tert-butoxy)carbonyl]amino}-4-{[(2 S)-1-[4-(3-hydroxyphenyl)piperidin-1-yl]-3-methylbutan-2-yl]carbamoyl}butyl]amino}({[(tert-butoxy)carbonyl]imino})methyl]carbamate (**II-2**)

Prepared according to General Procedure 3 Method A using (S)-3-(1-(2-amino-3-methylbutyl)piperidin-4-yl)phenol (**I-4**)^15^ (100 mg, 0.38 mmol) and Boc-Arg(Boc)_2_-OH (217 mg, 0.46 mmol) to afford 260 mg of the title material in 95% yield. ^1^H NMR (300 MHz, CDCl_3_) *δ* 9.40 (1 H, s), 7.14 (1 H, t, *J* = 5.7 Hz), 6.76 (1 H, s), 6.69 (2 H, t, *J* = 5.7 Hz), 6.19 (1 H, d, *J* = 6 Hz), 5.45 (1 H, d, J = 6.3 Hz), 4.15 (1 H, q, *J* = 11.1, 6 Hz), 3.86–3.99 (3 H, m), 2.91 (2 H, dd, *J* = 33.9, 25.8 Hz), 2.32–2.44 (3 H, m), 2.18 (2 H, t, *J* = 6.6 Hz), 1.60–1.90 (10 H, m), 1.46 (27 H, app t, *J* = 13.2 Hz), 0.92 (6 H, app t, *J* = 4.5 Hz). MS(ESI) *m/z* 719.44 (M + H)^+^.

### (S)-2-amino-5-guanidino-N-((S)-1-(4-(3-hydroxyphenyl)piperidin-1-yl)-3-methylbutan-2-yl)pentanamide (**7**)

Prepared according to General Procedure 4 Method A from intermediate **II-2** (250 mg, 0.35 mmol). The solution was concentrated, then triturated in diethyl ether overnight to afford 210 mg of the title material in 79% yield. A portion of the crude was purified via flash chromatography on a C18 reverse phase column (MeOH/H_2_O 100/0 → 75/25).^1^H NMR (300 MHz, MeOD-*d*_4_) *δ* 7.14 (1 H, t, *J* = 6 Hz), 6.65–6.73 (3 H, m), 4.18 (1 H, m), 3.96–4.00 (1 H, m), 3.65 (2 H, dd, *J* = 33, 15 Hz), 3.39 (2 H, d, *J* = 3 Hz), 3.08–3.27 (4 H, m), 2.82 (1 H, br s), 1.88–2.10 (7 H, m), 1.69–1.76 (2 H, m), 1.01 (6 H, app dd, *J* = 9, 3 Hz). HRMS (ESI) Calcd for C_22_H_39_N_6_O_2_ (M + H)^+^ 419.3129; found 419.3131. LCMS R_T_ = 0.79 min; *m/z* (M + H)^+^  = 419.40.

### (R)-2-(tert-butoxycarbonyl)-7-((tert-butyldimethylsilyl)oxy)-1,2,3,4-tetrahydroisoquinoline-3-carboxylic acid (**III-1a**)

To a mixture of (3 R)-2-(tert-Butoxycarbonyl)-7-hydroxy-1,2,3,4-tetrahydroisoquinoli ne-3-carboxylic acid **I-1** (500 mg, 1.71 mmol, 1.00 equiv) in DMF(ah) (5.00 mL) was added TBDMS-Cl (770 mg, 5.11 mmol, 3.00 equiv) and imidazole (695 mg, 10.23 mmol, 6.00 equiv) under Ar(g), and the reaction was stirred at room temperature for 3 h. The reaction was diluted with EtOAc and H_2_O (aq). The layers were separated, and the aqueous solution was extracted 2X with EtOAc. The combined organic layers were washed 3X with H_2_O then satd. NaCl(aq), dried over Na_2_SO_4_, filtered and concentrated. The residue was purified by flash chromatography using Hexanes/EtOAc 90/10 → 60/40 as the eluent to afford 568 mg of the title material in 82% yield. ^1^H NMR (300 MHz, CDCl_3_) *δ* 7.00 (1 H, d, *J* = 6 Hz), 6.65 (1 H, d, *J* = 6.6 Hz), 6.58 (1 H, s), 4.58–4.72 (2 H, m), 4.37–4.44 (1 H, m), 3.08–3.16 (2 H, m), 1.47 (9 H, d, *J* = 27.6 Hz), 0.97 (9 H, s), 0.18 (6 H, s). MS(APCI) *m/z* 308.1 (M + H-Boc)^+^.

### tert-butyl (R)-7-((tert-butyldimethylsilyl)oxy)-3-(((S)-1-(4-(3-hydroxyphenyl)piperidin-1-yl)-3-methylbutan-2-yl)carbamoyl)-3,4-dihydroisoquinoline-2(1 H)-carboxylate (**III-2a**)

Prepared according to General Procedure 3 Method A using (S)-3-(1-(2-amino-3-methylbutyl)piperidin-4-yl)phenol (**I-4**)^15^ (295 mg, 1.13 mmol) and intermediate **III-1a** (550 mg, 1.35 mmol) to afford 640 mg of the title material in 87% yield. ^1^H NMR (300 MHz, CDCl_3_) *δ* 7.14 (1 H, t, *J* = 9 Hz), 7.05 (1 H, d, *J* = 6 Hz), 6.66–6.72 (4 H, m), 6.60 (1 H, s), 6.34 (1 H, s), 5.98 (1 H, s), 4.79–4.82 (1 H, br s), 4.48–4.63 (2 H, m), 3.89 (1 H, br s), 3.04 (1 H, dd, *J* = 15,6 Hz), 1.66–2.79 (13 H, m), 1.51 (9 H, s), 0.96 (9 H, s), 0.87 (6 H, app dd, *J* = 18,9 Hz), 0.17 (6 H, s). MS(ESI) *m/z* 652 (M + H)^+^.

### tert-butyl (R)-7-((tert-butyldimethylsilyl)oxy)-3-(((S)-3-methyl-1-(4-(3-(((trifluoromethyl)sulfonyl)oxy)phenyl)piperidin-1-yl)butan-2-yl)carbamoyl)-3,4-dihydroisoquinoline-2(1 H)-carboxylate (**III-3a**)

Prepared according to General Procedure 1 using intermediate **III-2a** (430 mg, 0.66 mmol) to afford 320 mg of the title material in 62% yield. ^1^H NMR (300 MHz, CDCl_3_) *δ* 7.37 (1 H, t, *J* = 9 Hz), 7.22 (1 H, d, *J* = 9 Hz), 7.04–7.11 (3 H, m), 6.67 (1 H, dd, *J* = 9,3 Hz), 6.61 (1 H, br s), 5.85 (1 H, br s), 4.40–4.76 (3 H, m), 3.82 (1 H, br s), 3.28 (1 H, dd, *J* = 15,3 Hz), 3.00 (1 H, dd, *J* = 15,9 Hz), 2.71–2.82 (2 H, m), 2.36–2.50 (1 H, m), 1.88–2.22 (5 H, m), 1.64–1.72 (4 H, m), 1.51 (9 H, s), 0.94 (9 H, s), 0.85 (6 H, app dd, *J* = 21, 6 Hz), 0.14 (6 H, s). MS(ESI) *m/z* 784.8 (M + H)^+^.

### tert-butyl (R)-3-(((S)-3-methyl-1-(4-(3-(((trifluoromethyl)sulfonyl)oxy)phenyl)piperidin-1-yl)butan-2-yl)carbamoyl)-3,4-dihydroisoquinoline-2(1 H)-carboxylate (**III-3b**)

Prepared according to General Procedure 1 using intermediate **III-2b** (670 mg, 1.28 mmol) to afford 420 mg of the title material in 50% yield. ^1^H NMR (300 MHz, MeOD-*d*_4_) *δ* 7.45 (1 H, t, *J* = 6 Hz), 7.32 (1 H, d, *J* = 5.4 Hz), 7.18–7.19 (6 H, m), 4.80–4.83 (2 H, m), 4.62 (2 H, d, *J* = 9.3 Hz), 3.85 (1 H, s), 3.17–3.26 (3 H, m), 2.85 (2 H, s), 2.41–2.53 (3 H, m), 1.70–1.74 (5 H, m), 1.51 (9 H, s), 0.83 (6 H, s). MS(ESI) *m/z* 654 (M + H)^+^.

### tert-butyl (R)-7-((tert-butyldimethylsilyl)oxy)-3-(((S)-1-(4-(3-cyanophenyl)piperidin-1-yl)-3-methylbutan-2-yl)carbamoyl)-3,4-dihydroisoquinoline-2(1 H)-carboxylate (**III-4a**)

Prepared according to General Procedure 2 using intermediate **III-3a** (250 mg, 0.32 mmol) to afford 210 mg of the title material in 67% yield. ^1^H NMR (300 MHz, CDCl_3_) *δ* 7.47–7.50 (2 H, m), 7.39–7.41 (2 H, m), 7.06 (1 H, d, *J* = 9 Hz), 6.68 (1 H, dd, *J* = 9,3 Hz), 6.60 (1 H, br s), 5.80 (1 H, br s), 4.40–4.79 (3 H, m), 3.81 (1 H, br s), 3.29 (1 H, dd, *J* = 15, 3 Hz), 3.01 (1 H, dd, *J* = 12, 3 Hz), 2.72–2.82 (2 H, m), 2.37–2.47 (1 H, m), 1.87–2.19 (5 H, m), 1.63–1.72 (4 H, m), 1.52 (9 H, s), 0.94 (9 H, s), 0.85 (6 H, app dd, *J* = 21, 9 Hz), 0.15 (6 H, s). MS(ESI) *m/z* 661.5 (M + H)^+^.

### (R)-N-((S)-1-(4-(3-cyanophenyl)piperidin-1-yl)-3-methylbutan-2-yl)-7-hydroxy-1,2,3,4-tetrahydroisoquinoline-3-carboxamide (**8**)

Intermediate **III-4a** (202 mg, 0.31 mmol) was subjected to conditions described in General Procedure 6 to afford 170 mg of tert-butyl (R)-3-(((S)-1-(4-(3-cyanophenyl)piperidin-1-yl)-3-methylbutan-2-yl)carbamoyl)-7-hydroxy-3,4-dihydroisoquinoline-2(1 H)-carboxylate in quantitative yield. ^1^H NMR (300 MHz, MeOD-*d*_4_) *δ* 7.43–7.57 (4 H, m), 6.97 (1 H, d, *J* = 6 Hz), 6.60–6.62 (2 H, m), 4.72 (1 H, br s), 4.52 (2 H, br s), 3.83 (1 H, br s), 3.00–3.18 (2 H, m), 2.82 (2 H, br s), 2.36–2.51 (3 H, m), 1.99–2.09 (2 H, m), 1.62–1.74 (5 H, m), 1.50 (9 H, s), 0.83 (6 H, d, *J* = 6 Hz). MS(ESI) *m/z* 547.2 (M + H)^+^. The title material was prepared according to General Procedure 4 Method A from the Boc-protected intermediate (55 mg, 0.101 mmol). The solution was concentrated to dryness, then partitioned between CH_2_Cl_2_ and satd. NaHCO_3_ (aq). The layers were separated, and the aqueous solution was extracted 2X with CH_2_Cl_2_. The combined organic layers were dried over Na_2_SO_4_, filtered and concentrated. The residue was purified via flash chromatography using CH_2_Cl_2_/iPrOH/NH_4_OH(aq) 100/0/0 → 93/6/1 as the eluent to afford 26 mg of the title material in 58% yield, which was converted to the HCl salt via addition of 2 M HCl/ether. ^1^H NMR (300 MHz, MeOD-*d*_4_) *δ* 7.43–7.58 (4 H, m), 6.95(1 H, d, *J* = 9 Hz), 6.60 (1 H, dd, *J* = 9, 3 Hz), 6.51 (1 H, d, *J* = 3 Hz), 3.90–4.06 (3 H, m), 3.60 (1 H, dd, *J* = 9, 6 Hz), 3.11 (1 H, d, *J* = 12 Hz), 2.81–3.01 (3 H, m), 2.55–2.63 (1 H, m), 2.49 (2 H, d, *J* = 6 Hz), 2.21 (1 H, td, *J* = 9, 6 Hz), 2.07 (1 H, td, *J* = 12,3 Hz), 1.66–1.91 (5 H, m), 0.94 (6 H, app t, *J* = 6 Hz). MS(ESI) *m/z* 447.3 (M + H)^+^. Anal. Calcd. for C_27_H_34_N_4_O_2_^·^2.00 HCl^.^1.7 H_2_O^·^0.2 Diethyl Ether: C, 59.10; H, 7.39; N, 9.92; found: C, 59.20; H, 7.25; N, 9.76.

### (R)-N-((S)-1-(4-(3-(aminomethyl)phenyl)piperidin-1-yl)-3-methylbutan-2-yl)-7-hydroxy-1,2,3,4-tetrahydroisoquinoline-3-carboxamide (**10**)

Prepared according to General Procedure 5 from **8** (90 mg, 0.20 mmol) to afford 80 mg of the title material in 63% yield, which was converted to the HCl salt via addition of 2 M HCl/ether. ^1^H NMR (HCl salt, 300 MHz, MeOD-*d*_4_) *δ* 7.32–7.43 (4 H, m), 7.11 (1 H, d, *J* = 9 Hz), 6.76 (1 H, dd, *J* = 9,3 Hz), 6.66 (1 H, s), 4.32–4.46 (4 H, m), 4.12 (2 H, s), 3.65 (1 H, d, *J* = 12 Hz), 3.35–3.44 (1 H, m), 3.25–3.27 (3 H, m), 3.07–3.17 (3 H, m), 2.96 (1 H, t, *J* = 12 Hz), 2.62 (1 H, dd, *J* = 15, 3 Hz), 2.37 (1 H, dd, *J* = 12,3 Hz), 1.99–2.07 (2 H, m), 1.89 (1 H, q, *J* = 6 Hz), 1.03 (6 H, app t, *J* = 6 Hz). HRMS(ESI) Calcd. for C_27_H_39_N_4_O_2_ [M + H]^+^ 451.3067; Found 451.3068. LCMS R_T_ = 1.35 min; *m/z* (M + H)^+^  = 451.2.

### tert-butyl (R)-3-(((S)-1-(4-(3-cyanophenyl)piperidin-1-yl)-3-methylbutan-2-yl)carbamoyl)-3,4-dihydroisoquinoline-2(1 H)-carboxylate (**III-4b**)

Prepared according to General Procedure 2 using intermediate **III-3b** (230 mg, 0.35 mmol) to afford 170 mg of the title material in 91% yield. ^1^H NMR (300 MHz, CDCl_3_) *δ* 7.39–7.52 (4 H, m), 7.14–7.21 (4 H, m), 5.85 (1 H, br s), 4.83–5.00 (1 H, m), 4.52–4.72 (2 H, m), 3.87 (1 H, br s), 3.41 (1 H, dd, *J* = 15.6, 3.6 Hz), 3.08 (1 H, dd, *J* = 15, 6 Hz), 2.65–2.90 (2 H, m), 1.95–2.50 (4 H, m), 1.82–1.93 (3 H, m), 1.54–1.75 (11 H, m), 0.88 (6 H, app dd, *J* = 19.5, 6.9 Hz). MS(ESI) *m/z* 531 (M + H)^+^.

### (R)-N-((S)-1-(4-(3-cyanophenyl)piperidin-1-yl)-3-methylbutan-2-yl)-1,2,3,4-tetrahydroisoquinoline-3-carboxamide (**9**)

Prepared according to General Procedure 4 Method A from intermediate **III-4b** (170 mg, 0.32 mmol). The solution was concentrated to dryness, then partitioned between CH_2_Cl_2_ and satd. NaHCO_3_(aq). Solid NaCl was added. The layers were separated, and the aqueous solution was extracted 2X with CH_2_Cl_2_. The combined organic layers were dried over Na_2_SO_4_, filtered and concentrated. The crude residue was purified via flash chromatography using Hexane/EtOAc/NH_4_OH(aq) 90/10/1 → 10/90/1 as the eluent to afford 120 mg of the title material in 87% yield, which was converted to the HCl salt by addition of 2 M HCl/ether. ^1^H NMR (300 MHz, MeOD-*d*_4_) *δ* 7.53–7.56 (3 H, m), 7.43–7.48 (1 H, m), 7.14 (3 H, d, J = 3 Hz), 7.07 (1 H, br s), 3.97–4.14 (3 H, m), 3.63–3.68 (1 H, m), 3.10–3.14 (1 H, m), 2.93–3.03 (3 H, m), 2.48–2.63 (3 H, m), 2.22 (1 H, td, J = 12, 3 Hz), 2.06 (1 H, td, J = 12, 3 Hz), 1.63–1.89 (5 H, m), 0.95 (6 H, app t, J = 6 Hz). HRMS (ESI) Calcd for C_27_H_35_N_4_O (M + H)^+^ 431.2805; found 431.2824. LCMS R_T_ = 0.97 min; *m/z* (M + H)^+^  = 431.5.

### tert-butyl (R)-3-(((S)-1-(4-(3-(aminomethyl)phenyl)piperidin-1-yl)-3-methylbutan-2-yl)carbamoyl)-7-((tert-butyldimethylsilyl)oxy)-3,4-dihydroisoquinoline-2(1 H)-carboxylate (**III-5a**)

Prepared according to General Procedure 5 from intermediate **III-4a** (583 mg, 0.88 mmol) to afford 375 mg of the title material in 64% yield. ^1^H NMR (300 MHz, MeOD-*d*_4_) *δ* 7.03–7.28 (6 H, m), 6.67–6.69 (1 H, m), 4.75 (1 H, br s), 4.54 (2 H, br s), 3.83 (1 H, br s), 3.78 (2 H, s), 3.04–3.21 (2 H, m), 2.81–2.84 (2 H, m), 2.31–2.46 (3 H, m), 2.01 (2 H, br s), 1.66–1.83 (6 H, m), 1.51 (9 H, s), 0.97 (9 H, s), 0.81 (6 H, br s), 0.17 (6 H, app d, *J* = 3 Hz). MS(ESI) *m/z* 665.5 (M + H)^+^.

### tert-butyl (R)-3-(((S)-1-(4-(3-(aminomethyl)phenyl)piperidin-1-yl)-3-methylbutan-2-yl)carbamoyl)-3,4-dihydroisoquinoline-2(1 H)-carboxylate (**III-5b**)

Prepared according to General Procedure 5 using intermediate **III-4b** (286 mg, 0.54 mmol) to afford 413 mg of the title material in 70% yield. ^1^H NMR (300 MHz, CDCl_3_) *δ* 7.06–7.31 (8 H, m), 5.95 (1 H, s), 4.79–5.00 (1 H, m), 4.53–4.72 (2 H, m), 3.88 (2 H, br s), 3.39 (1 H, dd, *J* = 15.6, 3.3 Hz), 3.05–3.10 (1 H, m), 2.68–2.79 (2 H, m), 2.37–2.40 (1 H, m), 2.00–2.30 (2 H, br s), 1.87–1.93 (2 H, m), 1.60–1.77 (6 H, m), 1.54 (9 H, s), 0.88 (6 H, app dd, *J* = 17.4, 6.6 Hz). MS(ESI) *m/z* 535 (M + H)^+^.

### (R)-N-((S)-1-(4-(3-(aminomethyl)phenyl)piperidin-1-yl)-3-methylbutan-2-yl)-1,2,3,4-tetrahydroisoquinoline-3-carboxamide (**11**)

To a solution of intermediate **III-5b** (76 mg, 0.14 mmol) in MeOH (1.00 mL) was added 2 M HCl in diethyl ether (1.00 mL), and the reaction was stirred at room temperature for 19 h. The reaction was concentrated, then triturated 3X with diethyl ether to afford 69 mg of the title material as a 3 HCl salt in 89% yield. ^1^H NMR (300 MHz, MeOD-*d*_4_) *δ* 7.27–7.44 (8 H, m), 4.48 (2 H, s), 4.32–4.45 (2 H, m), 4.13–4.16 (3 H, m), 3.64–3.68 (1 H, m), 3.34–3.46 (4 H, m), 3.10–3.26 (2 H, m), 2.95–3.03 (1 H, m), 2.60–2.64 (1 H, m), 2.36–2.40 (1 H, m), 2.01–2.09 (2 H, m), 1.89–1.95 (1 H, m), 1.03–1.07 (6 H, m). MS(ESI) *m/z* 435 (M + H)^+^. Anal. Calcd. for C_27_H_38_N_4_O^.^3.00 HCl^.^0.7 H_2_O: C, 58.26; H, 7.68; N, 10.07; found: C, 57.94; H, 7.31; N, 9.91.

### tert-butyl (R)-3-(((S)-1-(4-(3-((S,E)-4,9-bis((tert-butoxycarbonyl)amino)-13,13-dimethyl-3,11-dioxo-12-oxa-2,8,10-triazatetradec-9-en-1-yl)phenyl)piperidin-1-yl)-3-methylbutan-2-yl)carbamoyl)-7-((tert-butyldimethylsilyl)oxy)-3,4-dihydroisoquinoline-2(1 H)-carboxylate (**III-6a**)

Prepared according to General Procedure 3 Method B from intermediate **III-5a** (370 mg, 0.56 mmol) to afford 360 mg of the title material in 58% yield. ^1^H NMR (300 MHz, MeOD-*d*_4_) *δ* 7.22 (1 H, t, *J* = 6 Hz), 7.03–7.12 (4 H, m), 6.66–6.68 (2 H, m), 4.73 (1 H, br s), 4.55 (1 H, br s), 4.37 (2 H, q, *J* = 15 Hz), 4.08 (1 H, br s), 3.85 (3 H, br s), 3.04–3.20 (2 H, m), 2.83 (2 H, br s), 1.93–2.52 (3 H, m), 1.64–1.71 (9 H, m), 1.43–1.52 (36 H, m), 0.96 (9 H, s), 0.81 (6 H, app d, *J* = 6 Hz), 0.16 (6 H, s). MS(ESI) *m/z* 1122.0 (M + H)^+^.

### tert-butyl (R)-3-(((S)-1-(4-(3-((S,E)-4,9-bis((tert-butoxycarbonyl)amino)-13,13-dimethyl-3,11-dioxo-12-oxa-2,8,10-triazatetradec-9-en-1-yl)phenyl)piperidin-1-yl)-3-methylbutan-2-yl)carbamoyl)-7-hydroxy-3,4-dihydroisoquinoline-2(1 H)-carboxylate (**III-7a**)

Prepared according to General Procedure 6 using intermediate **III-6a** (340 mg, 0.30 mmol) to afford 161 mg of the title material in 53% yield. ^1^H NMR (300 MHz, MeOD-*d*_4_) *δ* 7.22 (1 H, t, *J* = 6 Hz), 7.07–7.13 (3 H, m), 6.98 (1 H, d, *J* = 9 Hz), 6.60–6.63 (2 H, m), 4.71 (1 H, br s), 4.52 (2 H, br s), 4.37 (2 H, q, *J* = 15 Hz), 4.08 (1 H, br s), 3.84 (3 H, br s), 3.00–3.15 (2 H, m), 2.80 (2 H, br s), 1.93–2.39 (3 H, m), 1.64–1.79 (9 H, m), 1.43–1.52 (36 H, m), 0.83 (6 H, app d, *J* = 6 Hz). MS(ESI) *m/z* 1008.2 (M + H)^+^.

### tert-butyl (R)-3-(((S)-1-(4-(3-((S,E)-4,9-bis((tert-butoxycarbonyl)amino)-13,13-dimethyl-3,11-dioxo-12-oxa-2,8,10-triazatetradec-9-en-1-yl)phenyl)piperidin-1-yl)-3-methylbutan-2-yl)carbamoyl)-3,4-dihydroisoquinoline-2(1 H)-carboxylate (**III-6b**)

Prepared according to General Procedure 3 Method B from intermediate **III-5b** (120 mg, 0.22 mmol) to afford 102 mg of the title material in 46% yield. ^1^H NMR (300 MHz, CDCl_3_) *δ* 9.40 (1 H, s), 9.30 (1 H, s), 7.05–7.31 (8 H, m), 6.00–6.14 (1 H, m), 5.87 (1 H, br s), 4.65–4.93 (1 H, m), 4.36–4.58 (4 H, m), 3.82–3.93 (2 H, m), 3.64–3.74 (1 H, m), 3.36–3.41 (1 H, m), 3.06–3.10 (1 H, m), 2.64–2.74 (2 H, m), 2.32 (1 H, br s), 1.76–1.97 (4 H, m), 1.72 (5 H, br s), 1.53 (19 H, s), 1.46 (10 H, s), 1.39 (9 H, s), 0.87 (6 H, app dd, *J* = 19.8, 6.6 Hz). MS(ESI) *m/z* 992 (M + H)^+^.

### (R)-N-((S)-1-(4-(3-(((S)-2-amino-5-guanidinopentanamido)methyl)phenyl)piperidin-1-yl)-3-methylbutan-2-yl)-7-hydroxy-1,2,3,4-tetrahydroisoquinoline-3-carboxamide (**12**)

Prepared according to General Procedure 4 Method A from intermediate **III-7a** (86 mg, 0.09 mmol). The solution was concentrated, then triturated in ether overnight to afford 84 mg of the title material in 92% yield. ^1^H NMR (300 MHz, MeOD-*d*_4_) *δ* 7.31 (1 H, d, *J* = 6 Hz), 7.16–7.22 (3 H, m), 7.10 (1 H, d, *J* = 6 Hz), 6.76 (1 H, dd, *J* = 9, 3 Hz), 6.65 (1 H, s), 4.29–4.50 (6 H, m), 4.19 (1 H, dd, *J* = 12, 6 Hz), 4.07 (1 H, br s), 3.89 (1 H, t, *J* = 9 Hz), 3.63–3.66 (1 H, m), 3.06–3.23 (7 H, m), 2.86 (1 H, br s), 2.06–2.16 (4 H, m), 1.86–1.96 (3 H, m), 1.61–1.71 (2 H, m), 1.02 (6 H, app t, *J* = 6 Hz). HRMS (ESI) Calcd for C_33_H_51_N_8_O_3_ (M + H)^+^ 607.4079; found 607.4052. LCMS R_T_ = 1.16 min; *m/z* (M + H)^+^  = 607.5.

### (R)-N-((S)-1-(4-(3-(((S)-2-amino-5-guanidinopentanamido)methyl)phenyl)piperidin-1-yl)-3-methylbutan-2-yl)-1,2,3,4-tetrahydroisoquinoline-3-carboxamide (**13**)

Prepared according to General Procedure 4 Method B from intermediate **III-6b** (60 mg, 0.06 mmol). The solution was concentrated, then azeotroped 3X with diethyl ether to afford 60 mg of the title material as a 4 TFA salt in 93% yield. ^1^H NMR (300 MHz, MeOD-*d*_4_) *δ* 7.16–7.34 (8 H, m), 4.31–4.51 (6 H, m), 4.25 (1 H, dd, *J* = 12, 6 Hz), 4.07 (1 H, br s), 3.89 (1 H, t, *J* = 9 Hz), 3.63–3.67 (1 H, m), 3.42 (2 H, dd, *J* = 15, 3 Hz), 3.18–3.27 (5 H, m), 3.07 (1 H, br s), 2.90 (1 H, br s), 1.84–2.22 (7 H, m), 1.61–1.71 (2 H, m), 1.03 (6 H, app t, *J* = 6 Hz). LCMS R_T_ = 1.20 min; *m/z* (M + H)^+^  = 591.1. HRMS (ESI) Calcd for C_33_H_51_N_8_O_2_ (M + H)^+^ 591.4129; found 591.4141.

### (S)-1-(4-cyclohexylpiperidin-1-yl)-3-methylbutan-2-amine (**IV-2**)

To a solution of intermediate **IV-1** (313 mg, 1.27 mmol) in MeOH (12.7 mL) was added PtO_2_ (50 wt% of substrate) and concd. HCl (aq) (2.00 mL, 25.4 mmol, 20.0 equiv), and the mixture was hydrogenated at 4 atm H_2_(g) for 2.5 h at room temperature. The reaction was filtered through a pad of Celite, concentrated, and azeotroped with MeOH to afford 420 mg of the title material as a 2 HCl salt in quantitative yield. ^1^H NMR (300 MHz, MeOD-*d*_4_) *δ* 3.71–3.82 (2 H, m), 3.56 (1 H, d, *J* = 12 Hz), 3.34–3.45 (2 H, m), 3.06–3.14 (1 H, m), 2.88–2.95 (1 H, m), 1.96–2.10 (3 H, m), 1.66–1.79 (7 H, m), 1.15–1.42 (5 H, m), 1.05–1.09 (6 H, m), 0.94–1.02 (2 H, m). MS(ESI) *m/z* 253.46 (M + H)^+^.

### (R)-N-((S)-1-(4-cyclohexylpiperidin-1-yl)-3-methylbutan-2-yl)-7-hydroxy-1,2,3,4-tetrahydroisoquinoline-3-carboxamide (**14**)

Intermediate **IV-2** (150 mg, 0.59 mmol) and (3 R)-2-(tert-Butoxycarbonyl)-7-hydroxy-1,2,3,4-tetrahydroisoquinoli ne-3-carboxylic acid **I-1** (209 mg, 0.71 mmol) were subjected to conditions described in General Procedure 3 Method A to afford 244 mg of tert-butyl (R)-3-(((S)-1-(4-cyclohexylpiperidin-1-yl)-3-methylbutan-2-yl)carbamoyl)-7-hydroxy-3,4-dihydroisoquinoline-2(1 H)-carboxylate in 78% yield. ^1^H NMR (400 MHz, CDCl_3_) *δ* 7.00 (1 H, d, *J* = 8 Hz), 6.64 (1 H, d, *J* = 8 Hz), 6.51 (1 H, br s), 6.17 (1 H, br s), 5.86 (1 H, br s), 4.92 (1 H, br s), 4.70 (1 H, br s), 4.55 (1 H, d, *J* = 16 Hz), 4.40 (1 H, d, *J* = 16 Hz), 3.77 (1 H, br s), 3.21–3.25 (1 H, m), 2.94–2.98 (1 H, m), 2.56–2.64 (2 H, m), 2.03–2.29 (2 H, m), 1.84–1.88 (2 H, m), 1.61–1.68 (6 H, m), 1.49 (12 H, br s), 0.77–1.26 (13 H, m). MS(ESI) *m/z* 528.78 (M + H)^+^. The title material was prepared according to General Procedure 4 Method C using Boc-protected intermediate (230 mg, 0.44 mmol). The crude residue was diluted with CH_2_Cl_2_ and satd. NaHCO_3_(aq). Solid NaCl was added. The layers were separated, and the aqueous solution was extracted 2X with CH_2_Cl_2_. The combined organic layers were washed with satd. NaCl(aq), dried over Na_2_SO_4_, filtered and concentrated. The aqueous layers were combined and extracted 2X with EtOAc. The combined organic layers were washed with satd. NaCl(aq), dried over Na_2_SO_4_, filtered and concentrated. The crude residue was purified via flash chromatography using Hexane/EtOAc/iPrOH/NH_4_OH 50/50/0/0 → 42.5/42.5/15/1 to afford 146 mg of the title material in 78% yield, which was converted to the HCl salt by addition of 2 M HCl/ether. ^1^H NMR (300 MHz, MeOD-*d*_4_) *δ* 6.92 (1 H, d, *J* = 8 Hz), 6.58 (1 H, dd, *J* = 8, 4 Hz), 6.47–6.48 (1 H, m), 3.88–4.01 (3 H, m), 3.52–3.56 (1 H, m), 3.02 (1 H, d, *J* = 8 Hz), 2.89–2.94 (2 H, m), 2.76–2.83 (1 H, m), 2.42–2.45 (2 H, m), 2.02 (1 H, t, *J* = 8 Hz), 1.63–1.91 (10 H, m), 0.95–1.32 (8 H, m), 0.90 (6 H, app t, *J* = 8 Hz). MS(ESI) *m/z* 428.4 (M + H)^+^. Anal. Calcd. for C_26_H_41_N_3_O_2_^.^2.00 HCl^.^1.00 H_2_O: C, 60.22; H, 8.75; N, 8.10; found: C, 60.46; H, 8.51; N, 7.89.

### (S)-1-(1-(2-amino-3-methylbutyl)piperidin-4-yl)indolin-2-one (**V-4**)

To a mixture of N-(4-piperidyl)-1,3-dihydroindol-2-one **V-1** (510 mg, 2.36 mmol, 1.00 equiv)^31^ in 1,2-dichloroethane (DCE) (39.0 mL) was added Boc-L-valinal **V-3** (754 mg, 3.75 mmol, 1.60 equiv) and HOAc (0.20 mL, 3.53 mmol, 1.50 equiv), and the reaction was stirred at room temperature for 0.5 h. NaBH(OAc)_3_ (750 mg, 3.54 mmol, 1.50 equiv) was added, and the reaction was stirred at room temperature for 16 h. The reaction was diluted with CH_2_Cl_2_ and satd. NaHCO_3_(aq). Solid NaHCO_3_ was added. The layers were separated, and the aqueous solution was extracted 2X with CH_2_Cl_2_. The combined organic layers were washed with satd. NaCl(aq), dried over Na_2_SO_4_, filtered and concentrated. The crude residue was purified via flash chromatography with flash chromatography with Hexane/EtOAc/NH_4_OH(aq) 89/10/1 → 30/69/1 to afford 575 mg of tert-butyl (S)-(3-methyl-1-(4-(2-oxoindolin-1-yl)piperidin-1-yl)butan-2-yl)carbamate in 61% yield. ^1^H NMR (400 MHz, CDCl_3_) *δ* 7.21 (2 H, t, *J* = 4 Hz), 7.09 (1 H, d, *J* = 8 Hz), 6.99 (1 H, t, *J* = 8 Hz), 4.51 (1 H, s), 4.25–4.29 (1 H, m), 3.48 (2 H, s), 3.00 (2 H, dd, *J* = 52, 12 Hz), 2.31–2.45 (4 H, m), 2.21 (1 H, t, *J* = 12 Hz), 2.02 (1 H, t, *J* = 12 Hz), 1.85–1.89 (1 H, m), 1.75 (1 H, s), 1.65 (2 H, d, *J* = 12 Hz), 1.45 (9 H, s), 0.89 (6 H, app dd, *J* = 24, 8 Hz). MS(ESI) *m/z* 402.4 (M + H)^+^. The title material was prepared according to General Procedure 4 Method B using the Boc-protected intermediate (585 mg, 1.46 mmol). The solution was concentrated, then azeotroped 3X with CH_2_Cl_2_ to afford 771 mg of the title material as a 2 TFA salt in quantitative yield, which was used directly in the next reaction. MS(ESI) *m/z* 302.3 (M + H)^+^.

### (S)-1-(4-(indolin-1-yl)piperidin-1-yl)-3-methylbutan-2-amine (**V-5**)

To a mixture of 1-(piperidin-4-yl)indoline 2,2,2-trifluoroacetate **V-2** (582 mg free base equivalent, 2.88 mmol, 1.00 equiv) in 1,2-dichloroethane (15.0 mL) was added Et_3_N (0.40 mL, 2.88 mmol, 1.00 equiv), and the reaction was stirred at room temperature for 5 min under Ar(g). To this mixture was added Boc-L-valinal **V-3** (3.00 g, 8.63 mmol, 3.00 equiv) in 1,2-dichloroethane (33.0 mL), followed by HOAc (0.49 mL, 8.63 mmol, 3.00 equiv), and the reaction was stirred at room temperature for 1 h. NaBH(OAc)_3_ (1.83 g, 8.63 mmol, 3.00 equiv) was added, and the reaction was stirred at room temperature for 16 h. The reaction was diluted with CH_2_Cl_2_ and satd. NaHCO_3_(aq). The layers were separated, and the aqueous solution was extracted 2X with CH_2_Cl_2_. The combined organic layers were washed with satd. NaCl(aq), dried over Na_2_SO_4_, filtered and concentrated. The crude residue was purified via flash chromatography using Hexane/EtOAc/NH_4_OH 90/10/0 → 40/60/1 to afford 690 mg of the iminium intermediate 1-((S)-2-((tert-butoxycarbonyl)amino)-3-methylbutylidene)-4-(indolin-1-yl)piperidin-1-ium in 62% yield. To a solution of iminium intermediate (630 mg, 1.63 mmol, 1.00 equiv) in trifluoroethanol (14.0 mL) was added NaCNBH_3_ (3.58 g, 57.0 mmol, 40.0 equiv), and the reaction was heated to 50 °C for 18 h. The reaction was diluted with EtOAc and satd. NaHCO_3_(aq). The layers were separated, and the aqueous solution was extracted 2X with EtOAc. The combined organic layers were washed with satd. NaCl(aq), dried over Na_2_SO_4_, filtered and concentrated to give 795 mg of crude tert-butyl (S)-(1-(4-(indolin-1-yl)piperidin-1-yl)-3-methylbutan-2-yl)carbamate in quantitative yield. ^1^H NMR (300 MHz, CDCl_3_) *δ* 7.02–7.07 (2 H, m), 6.62 (1 H, t, *J* = 6 Hz), 6.42 (1 H, d, *J* = 9 Hz), 4.67 (2 H, d, *J* = 9 Hz), 3.76 (2 H, dd, *J* = 9, 3 Hz), 3.35–3.58 (7 H, m), 2.96 (1 H, t, *J* = 6 Hz), 1.66–1.95 (5 H, m), 1.45–1.46 (9 H, m), 0.94 (6 H, app t, *J* = 6 Hz). MS(ESI) *m/z* 388.4 (M + H)^+^. The title material was prepared according to General Procedure 4 Method B from the Boc-protected intermediate (770 mg, 1.99 mmol). The solution was concentrated, then azeotroped 3X with MeOH and 3X with CH_2_Cl_2_ to afford 1.04 g of the title material as a 2 TFA salt in quantitative yield, which was used directly in the next reaction. MS(ESI) *m/z* 288.3 (M + H)^+^.

### (R)-7-hydroxy-N-((S)-3-methyl-1-(4-(2-oxoindolin-1-yl)piperidin-1-yl)butan-2-yl)-1,2,3,4-tetrahydroisoquinoline-3-carboxamide (**15**)

To a solution of intermediate **V-4** (771 mg, 1.46 mmol, 1.00 equiv) in 14.6 mL THF was added DiPEA (4.56 mL, 26.2 mmol, 18.0 equiv), and the mixture was stirred at room temperature for 10 min. (3 R)-2-(tert-Butoxycarbonyl)-7-hydroxy-1,2,3,4-tetrahydroisoquinoli ne-3-carboxylic acid **I-1** (470 mg, 1.60 mmol, 1.10 equiv) and propylphosphonic anhydride (T_3_P^®^, 50 wt% in EtOAc) (2.60 mL, 8.74 mmol, 6.00 equiv) was added, and the reaction was stirred at room temperature for 25 h. The reaction was diluted with CH_2_Cl_2_ and H_2_O. The layers were separated, and the aqueous solution was extracted 2X with CH_2_Cl_2_. The combined organic layers were washed with satd. NaCl(aq), dried over Na_2_SO_4_, filtered and concentrated. The crude residue was purified via flash chromatography with flash chromatography with Hexane/EtOAc/NH_4_OH(aq) 89/10/1 → 20/79/1 to afford 327 mg of tert-butyl (R)-7-hydroxy-3-(((S)-3-methyl-1-(4-(2-oxoindolin-1-yl)piperidin-1-yl)butan-2-yl)carbamoyl)-3,4-dihydroisoquinoline-2(1 H)-carboxylate in 39% yield. ^1^H NMR (400 MHz, CDCl_3_) *δ* 7.19–7.24 (2 H, m), 6.97–7.04 (3 H, m), 6.66 (2 H, dd, *J* = 8, 4 Hz), 4.01 (1 H, br s), 3.76–3.82 (3 H, m), 3.54 (2 H, s), 3.34 (1 H, d, *J* = 16 Hz), 3.14–3.20 (1 H, m), 2.85–3.01 (2 H, m), 2.69–2.71 (2 H, m), 2.00–2.46 (5 H, m), 1.84 (1 H, br s), 1.65 (2 H, br s), 1.50 (9 H, s), 0.83 (6 H, app dd, *J* = 16, 8 Hz). MS(ESI) *m/z* 577.5 (M + H)^+^. The title material was prepared according to General Procedure 4 Method C from the Boc-protected intermediate (102 mg, 0.18 mmol). The crude residue was azeotroped 3X with MeOH and 3X with CH_2_Cl_2_ to afford 95 mg of the title material as a 2 HCl salt in 98% yield. ^1^H NMR (free base, 400 MHz, CDCl_3_) *δ* 7.15–7.23 (2 H, m), 7.04–7.08 (1 H, m), 6.94–7.01 (2 H, m), 6.53–6.67 (2 H, m), 4.17–4.35 (1 H, m), 4.04–4.12 (1 H, m), 3.62–3.89 (5 H, m), 3.52–3.57 (1 H, m), 3.50 (2 H, s), 3.37–3.42 (1 H, m), 3.23–3.26 (1 H, m), 2.99–3.13 (2 H, m), 2.28–2.85 (4 H, m), 2.07–2.21 (1 H, m), 1.82–1.93 (1 H, m), 1.64–1.71 (2 H, m), 0.88–0.96 (6 H, m). HRMS (ESI) Calcd for C_28_H_37_N_4_O_3_ (M + H)^+^ 477.2860; found 477.2860. LCMS R_T_ = 1.21 min; *m/z* (M + H)^+^  = 477.4.

### (R)-7-hydroxy-N-((S)-1-(4-(indolin-1-yl)piperidin-1-yl)-3-methylbutan-2-yl)-1,2,3,4-tetrahydroisoquinoline-3-carboxamide (**16**)

Intermediate **V-5** (392 mg, 1.36 mmol) and (3 R)-2-(tert-Butoxycarbonyl)-7-hydroxy-1,2,3,4-tetrahydroisoquinoli ne-3-carboxylic acid (**I-1**) (480 mg, 1.64 mmol) were subjected to conditions described in General Procedure 3 Method A to afford 219 mg of tert-butyl (R)-7-hydroxy-3-(((S)-1-(4-(indolin-1-yl)piperidin-1-yl)-3-methylbutan-2-yl)carbamoyl)-3,4-dihydroisoquinoline-2(1 H)-carboxylate in 29% yield. ^1^H NMR (300 MHz, CDCl_3_) *δ* 6.98–7.07 (3 H, m), 6.60–6.68 (3 H, m), 6.40 (1 H, d, *J* = 9 Hz), 4.80 (1 H, br s), 4.49 (1 H, br s), 3.86 (1 H, br s), 3.22–3.35 (5 H, m), 2.91–3.00 (5 H, m), 1.97–2.49 (3 H, br s), 1.65–1.87 (5 H, m), 1.51 (9 H, s), 0.81–0.89 (6 H, m). MS(ESI) *m/z* 563.5 (M + H)^+^. The title material was prepared according to General Procedure 4 Method C from the Boc-protected intermediate (45 mg, 0.08 mmol). The crude residue was triturated with dioxane to afford 34 mg of the title material as a 2 HCl salt in 79% yield. ^1^H NMR (300 MHz, MeOD-*d*_4_) *δ* 7.08–7.36 (5 H, m), 6.75 (1 H, dd, *J* = 6, 3 Hz), 6.66 (1 H, d, *J* = 3 Hz), 4.12–4.36 (5 H, m), 3.83 (1 H, t, *J* = 9 Hz), 3.68 (3 H, br s), 3.34–3.40 (2 H, m), 3.05–3.23 (6 H, m), 2.69–2.85 (1 H, m), 2.41–2.52 (1 H, m), 2.01–2.11 (2 H, m), 1.83–1.90 (1 H, m), 1.01 (6 H, app t, *J* = 6 Hz). LCMS R_T_ = 1.29 min; *m/z* (M + H)^+^  = 463.3. HRMS (ESI) Calcd for C_28_H_39_N_4_O_2_ (M + H)^+^ 463.3067; found 463.3096.

### *In vitro* pharmacological Characterization

#### Cells

Human NOP, mu, delta, and kappa opioid receptors were individually expressed in Chinese hamster ovary cells stably transfected with the human receptor cDNA, as we have described previously^2,38^. Kappa-CN cells were used for KOP radioligand binding assays, while Kappa-FLG19 cells were used in KOP [^35^S]GTPγS functional assays. The cells were grown in Dulbecco’s Modified Eagle Medium (DMEM) with 10% fetal bovine serum, in the presence of 0.4 mg/ml G418 and 0.1% penicillin/streptomycin, in 100-mm plastic culture dishes.

#### Membrane preparation

The cell lines are grown to full confluency, then harvested for membrane preparation. The membranes are prepared in 50 mM Tris buffer (pH 7.4). Cells are scraped and centrifuged at 500 × g for 12 mins. The cell pellet is homogenized in 50 mM Tris with a Fisher Scientific PowerGen 125 rotor-stator type homogenizer, centrifuged at 20,000 × g for 25 mins, washed and recentrifuged once more and aliquoted at a concentration of 3 mg/mL protein per vial and stored in a −80 °C freezer till further use.

#### Receptor Binding

The assay is performed in a 96–well polystyrene plate using triplicates of six concentrations of each test compound and tritiated ligands [^3^H]DAMGO (0.2 nM for MOP), [^3^H]DPDPE (0.2 nM for DOP), [^3^H]U69593 (0.2 nM for KOP), or [^3^H]N/OFQ (0.2 nM for NOP). Nonspecific binding was determined by using 1.0 µM of the unlabeled nociceptin for NOP, 10 µM unlabeled DAMGO for MOP, 10 µM unlabeled DPDPE for DOP, and 10 µM unlabeled U69,593 for KOP. Assays were initiated by addition of membrane homogenates and samples were incubated for 60 min at 25 °C in a total volume of 1.0 mL. In NOP receptor experiments, 1 mg/mL BSA is added to the assay buffer. The amount of protein in the binding assay was 15 μg. The incubation was terminated by rapid filtration through 0.5% PEI-soaked glassfiber filter mats (GF/C Filtermat A, Perkin-Elmer) on a Tomtec Mach III cell harvester and washed 5 times with 0.5 mL of ice-cold 50 nM Tris-HCl, pH 7.4 buffer. The filters were dried overnight and soaked with scintillation cocktail before counting on a Wallac Beta plate 1205 liquid scintillation counter. Radioactivity was determined as counts per minutes (cpm). Full characterization of compounds includes analysis of the data for IC_50_ values and Hill coefficients using GraphPad Prism. (ISI, San Diego, CA). K_i_ values were determined by the method of Cheng and Prusoff^39^.

#### [^35^S]GTPγS Functional assay

Functional assay is conducted in Buffer A, containing 20 mM HEPES, 10 mM MgCl_2_ 100 mM NaCl at pH 7.4. Membrane prepared as described above was incubated with [^35^S]GTPγS (150,000 dpm/well), GDP (10 μM), and the test compound, in a total volume of 1 mL, for 120 minutes at 25 °C. Samples were filtered over Filtermat A and counted as described for the binding assays. A dose response curve with a prototypical full agonist at the respective receptor is conducted in each experiment to identify full and partial agonist compounds.

#### Determination of Antagonist potency

High affinity compounds (K_i_ value < 50 nM) that demonstrate no agonist activity were evaluated for their antagonist potency by Schild analysis^40^, using an agonist full dose response curve in the presence of at least three concentrations of the test antagonist. pA_2_ values and Schild slopes are determined using a statistical program designed for these experiments. If the Schild slope was found to be significantly different from -1.00, the antagonist activity was deemed non-competitive; in such cases, the pA_2_ value is not reported. Equilibrium dissociation constants (K_e_ values) were calculated as follows:

$${{\rm{K}}}_{{\rm{e}}}=a/(DR-1)$$

where “a” is the nanomolar concentration of the antagonist and “DR” is the ratio of the agonist EC_50_ in the presence of a given concentration of antagonist.

### Molecular Docking

Compounds were sketched and minimized using MMFF94 force field and charges in SybylX 1.2. Molecular docking was carried out using the Surflex-dock module in SybylX 1.2. The protomol was defined using the existing ligand JDTic inside the KOP receptor binding site of the KOP crystal structure (PDB ID: 4DJH). Docking was performed using the Geom protocol in Surflex-dock. A total of 20 poses were retained for each molecule. The top scoring poses of **14** and **16** were analyzed and compared to the bound ligand JDTic.

### Data availability

The authors declare that all data supporting the findings of this study are available within the article.
